# Would you like to add a weight after this blood pressure, doctor? Discovery of potentially actionable associations between the provision of multiple screens in primary care

**DOI:** 10.1111/jep.12877

**Published:** 2018-01-19

**Authors:** Sumeet Kalia, Michelle Greiver, Xu Zhao, Christopher Meaney, Rahim Moineddin, Babak Aliarzadeh, Eva Grunfeld, Frank Sullivan

**Affiliations:** ^1^ Department of Family and Community Medicine University of Toronto Toronto Ontario Canada; ^2^ North York General Hospital Toronto Ontario Canada; ^3^ Dalla Lana School of Public Health Toronto Ontario Canada; ^4^ University of St Andrews St Andrews Fife Scotland, UK

**Keywords:** healthcare, patient‐centered care, person‐centered medicine, public health

## Abstract

**Rationale, aims, and objective:**

Guidelines recommend screening for risk factors associated with chronic diseases but current electronic prompts have limited effects. Our objective was to discover and rank associations between the presence of screens to plan more efficient prompts in primary care.

**Methods:**

Risk factors with the greatest impact on chronic diseases are associated with blood pressure, body mass index, waist circumference, glycaemic and lipid levels, smoking, alcohol use, diet, and exercise. We looked for associations between the presence of screens for these in electronic medical records. We used association rule mining to describe relationships among items, factor analysis to find latent categories, and Cronbach α to quantify consistency within latent categories.

**Results:**

Data from 92 140 patients in or around Toronto, Ontario, were included. We found positive correlations (lift >1) between the presence of all screens. The presence of any screen was associated with confidence greater than 80% that other data on items with high prevalence (blood pressure, glycaemic and lipid levels, or smoking) would also be present. A cluster of rules predicting the presence of blood pressure were ranked highest using measures of interestingness such as standardized lift. We found 3 latent categories using factor analysis; these were laboratory tests, vital signs, and lifestyle factors; Cronbach α ranged between .58 for lifestyle factors and .88 for laboratory tests.

**Conclusions:**

Associations between the provision of important screens can be discovered and ranked. Rules with promising combinations of associated screens could be used to implement data driven alerts.

## 1 INTRODUCTION

Chronic diseases are the leading causes of mortality and morbidity in upper middle and high‐income countries.^1^ It has been estimated that 40% to 70% of premature death and disability could be prevented through better control of risk factors associated with chronic conditions.^2^, ^3^ A small number of modifiable risk factors, including tobacco use, obesity, sedentary behaviour, increased blood glucose, and hypertension, account for most of the excess mortality and morbidity.^3^, ^4^, ^5^ Asking about and recording these risks are essential prerequisites to their monitoring and management.

The Canadian and US Task Forces on Preventive Health Care, as well as other guideline developers, have provided evidence‐based recommendations for chronic disease prevention, screening, and management (CDPSM).^6^, ^7^ These were recently reviewed and summarized as part of the BETTER trial,^8^ through evidence‐based reviews of multiple guidelines.^9^ Recommendations applicable to almost all patients age 45 or older and responsible for the largest effect on health included recording tobacco use, alcohol use, diet, exercise, fasting blood glucose or haemoglobin A1c (A1c), lipid profile including low‐density lipoprotein (LDL), body mass index (BMI), waist circumference (WC), and blood pressure (BP).^8^ While there is some controversy about the frequency with which these data elements should be recorded, frequently proposed intervals and standards for most patients are at least every 2 years for vital signs (BP, WC, and BMI) and at least every 3 years for laboratory tests (fasting blood glucose/A1c and LDL), recorded information about lifestyle risk factors in a summary health profile.^10^

However, physicians do not screen their patients consistently. For example, recording tobacco use in primary care electronic medical records (EMRs) has been found to be inconsistent and may vary by patient factors or physician characteristics.^11^, ^12^ A recent study found that only 64% of Canadian adults had a record of their smoking status in their EMR,^12^ and alcohol use was documented in only 20% of records.^13^

Patients often have multiple concurrent risk factors^14^; this increases mortality and morbidity beyond the sum of excess risk attributed to each individual factor.^15^ It is therefore important to screen for the presence and combination of multiple risk factors for each patient. A novel method to study this is to discover promising associations. This could be operationalized to increase the provision of multiple screens for each patient by leveraging data on associations between presence of screens.

There are several methods appropriate for the discovery of interesting associations in large data sets. Similar results obtained using different approaches would reinforce conclusions of associations between CDPSM items.

Association rule mining,^16^ which has also been called affinity analysis or market basket analysis, is a very commonly used approach to look for and identify interesting patterns in large databases containing many variables. This method has been successfully used in non‐medical domains such as marketing to understand and influence consumer behaviour^17^; the results lead to recommendations for products that a consumer may wish to purchase based on associations with current or prior purchases or peer purchasing behaviour.^18^ Association rule mining is a powerful technique for website design and is used to segment consumer groups for targeted marketing.^19^, ^20^, ^21^ Association rule mining has been used in large health care datasets for phenotype discovery and bioinformatics^22^, ^23^ and to study clustering of lifestyle choices and risk factors in patients.^14^ However, this method has not been used to study and affect the provision of multiple screens; large health care transactional databases such as those underlying EMRs may provide a rich source of information on associations between the provision of different screens and other health services.

Additional methods for association discovery include exploratory factor analysis; this is a multivariate statistical approach that can identify the underlying structure of groups of items.^24^ The consistency of latent variables discovered by factor analysis can be quantified using a psychometric measure such as Cronbach α.

Items that occur reasonably frequently are of particular interest.^16^ Reminders may be triggered too frequently when rare items are missing; this may lead to unintended consequences such as disregarding prompts.^25^ Frequent items are more likely to be actionable through targeted reminders. The screens included in this study are some of the most frequently recorded data in primary care because they are applicable to entire practice populations. As has been implemented in retail and marketing, information produced through the discovery of associations can point to the design of methods to influence the provision of multiple screens in primary care through targeted prompts and alerts based on available data patterns. However, information on associations between the provision of multiple screens is currently limited. In this project, we mine associations between the provision of screens that collectively account for the largest proportion of excess mortality and morbidity and that are recommended for the majority patients age 45 or older.

The objectives of this study were to discover, describe, and rank associations between sets of evidence‐based screens. We aim to enable the identification of associations that could be put into action to increase the proportion of eligible screens provided for each patient.

## 2 MATERIALS AND METHODS

### 2.1 Data sources and study population

This was a cross‐sectional study using routinely collected clinical EMR data. Eighty percent of Canadian family physicians reported using EMRs in 2014,^26^ making EMRs a good source of data about CDPSM items in Canadian primary care. We used data from the University of Toronto Practice Based Research Network (UTOPIAN) database. The UTOPIAN is one of 11 networks participating in the Canadian Primary Care Sentinel Surveillance Network (CPCSSN), Canada's EMR‐based chronic disease surveillance system.^27^, ^28^ Consenting family physicians in the Greater Toronto Area (Ontario, Canada) participating in UTOPIAN contribute deidentified EMR data to a data repository. This study includes data from 4 different EMR platforms. We included EMR data extracted as of June 30, 2015, using procedures previously described.^28^

The study population included individuals that were at least 45 years of age as of June 30, 2015, and had at least one encounter with their practice recorded in the EMR in the 2 years prior to the date of extraction; this visit interval has been used in other studies for primary care populations of interest.^29^, ^30^

This study was reviewed and approved by the Research Ethics Board at the North York General Hospital. All participating primary care providers have provided written informed consent for the collection and analysis of their EMR data.

### 2.2 Analytic approaches

We used proportions, standard deviations, and Venn diagrams to describe the data. Our analytic approaches included association rule mining, exploratory factor analysis, and Cronbach α.

### 2.3 Association rule mining

Association rule mining was first introduced by Agrawal et al in 1993 to discover associations in large transactional databases.^16^ We used association rules to examine relationships among nine CDPSM items. Association rules are expressed as A → B, where antecedent (A) and consequent (B) are collections of unique CDPSM items (*A* ∩ *B* = ∅). The implication sign (→) referred to the co‐occurrence of CDPSM items in the form of “if‐then” statement; this implies co‐occurrence among CDPSM items but not causal relationships. The strength of an association rule can be quantified using “support” and “confidence.”^17^ Support is defined as the *prevalence* of an item set: *support*(A → B) = Probability(A, B) while the confidence is the conditional probability that B will be present if A is present: *confidence*(A → B) = Probability(B|A).

Many algorithms including Apriori, ECLAT, FP‐growth, and LCM are available to efficiently mine frequent item sets.^31^ Given computational efficiency coupled with simplicity, we chose to use the Apriori algorithm^32^ in the “arules” package of R software (version 3.3.0) to generate the association rules.^33^ This algorithm allows specification of minimum support and minimum confidence prior to the generation of association rules. In this study, we specified the minimum support and confidence thresholds as 2% and 80%, respectively. The minimum support threshold removed the infrequent item sets (since these may be of less interest for the purposes of this study) while the minimum confidence threshold generated rules with strong associations.

A commonly used method to mine association rules is to rank measures of interestingness such as lift, leverage, Gini's index, or Yule's Q.^31^ Tan et al^34^ outline the properties of several interestingness measures and also provide some guidance on the selection of different measures. In this study, we used “Lift” as the general measure of association among CDPSM elements; this is defined as $\text{Lift}\;\left(A\to B\right)=\frac{\mathrm{Pr}\left(A,B\right)}{\mathrm{Pr}\left(A\right)\;\mathrm{Pr}\left(B\right)}$. Lift indicates the presence of several items together beyond chance. It is equal to one when A and B are statistically independent; it is greater than one when A and B are positively correlated and less than one when they are negatively correlated. Furthermore, lift has several desirable properties as noted by Shaikh et al.^35^ However, lift may perform poorly in the presence of random noise in transactional databases.^36^ Hence, we chose to standardize the lift with respect to its lower (*λ*) and upper (*ν*) bound^37^ as

$$L^{*}\left(A\to B\right)=\frac{L\left(A\;\to B\right)-\lambda }{\nu -\lambda }.$$

The upper bound (*ν*) is defined as the inverse of maximum probability of antecedent and consequent $\left(\nu =\frac{1}{\max\left(P\left(A\right),P\left(B\right)\right)}\right)\;$while the lower bound (*λ*) is defined as the maximization over four set:

$$\lambda =\text{max}\left\{\frac{P\left(A\right)+P\left(B\right)-1}{P\left(A\right)P\left(B\right)},\frac{4\sigma }{{\left(1+\sigma \right)}^{2}},\frac{\sigma }{P\left(A\right)P\left(B\right)},\frac{\;\kappa }{P\left(B\right)}\right\}.$$

The minimum support (2%) and confidence thresholds (80%) are denoted as *σ* and *κ*, respectively. The standardized lift ranged from zero to one, where one indicates the maximum value that the raw lift achieved for a particular association rule. The upper and lower bounds of lift are derived using Fréchet inequalities and are further discussed by Shaikh et al.^35^

Health data include many association rules with redundant items; dealing with a great number of rules is unnecessary and inefficient.^38^ Hence, in addition to ranking the interestingness measures, we removed rules containing redundant information. First, we formed clusters of association rules conditioned on the consequent and then we removed the rules containing redundant information from each cluster. Redundant rules were defined as rules that contained a subset of CDPSM items in relation to their super rule. As an example, consider the following 2 rules:

1. [A, B, C] → [D] and
2. [A, B] → [D].

Here, the second rule is redundant with respect to the first rule. Previously, McNicholas et al 37 used a similar mining strategy to extract the most useful information from large transactional databases. The grouping or clustering of association rules and subsequent pruning is an active area of research in data mining.^39^, ^40^

### 2.4 Factor analysis and Cronbach α

An assumption in large data sets is that there are underlying constructs or “latent” factors that represent relationships between items but are unmeasured and unobserved. Exploratory factor analysis can be used to discover those factors.^24^ We used factor analysis to represent 9 CDPSM items as a linear combination of 3 latent factors. We express the 3 factors as *f*_1_, *f*_2_, *f*_3_ and 9 CDPSM items as *y*_1_, *y*_2_, …*y*_9_. We assumed that the random sample of 9 CDPSM items was obtained from a homogeneous population with a mean vector denoted as *μ* and variance‐covariance matrix denoted as ∑. The mean vector *μ* corresponded to the frequency of recording nine CDPSM item while ∑ matrix described the variance‐covariance among 9 CDPSM items. Since the 9 CDPSM items were described as linear combination of 3 factors with accompanying error term, we partitioned the variance of 9 CDPSM items into communality and specific variance components. To identify latent grouping of 9 CDPSM items, we used oblique rotation which referred to a transformation where axes were not required to be perpendicular.

Once the CDPSM items were grouped, we then used Cronbach α to measure consistency within the 3 groups. Cronbach α is defined as $\alpha =\frac{k}{k-1}\;\left(1-\frac{\sum s_{i}^{2}}{s_{T}^{2}}\right)$, where *k* represents the total number of CDPSM items, $s_{i}^{2}$ is the variance of *i*th CDPSM item, and $\;s_{T}^{2}$ is the variance of the total score created by summing 9 CDPSM items. Cronbach α has a direct interpretation because the variance of the sum of 9 independent CDPSM items is the sum of their variances. Hence, Cronbach α is equal to one if CDPSM items are perfectly related with one another and zero if CDPSM items are not related with one another.

Factor analysis was conducted in R software (version 3.3.0; “psych” package) using the principle axis algorithm with “oblimin” rotation of 3 latent factors and assuming tetrachoric correlation among CDPSM elements.

## 3 RESULTS

Data were extracted from the EMRs of 180 primary care providers. The dataset included information on 92 140 patients age 45 or older. Table 1 provides information on patient and physician characteristics; Table 2 provides the frequency (or support) for each CDPSM item. A total of 3382 patients (3.7%) had no CDPSM item recorded, and 452 (0.5%) had all 3 items recorded.

**Table 1:** Patient and physician characteristics

Patient Characteristics	
Number of patients, n	92 140
Mean age, years (SD)	62.4 (12.4)
Male gender, n (%)	36 972 (40%)
At least one chronic condition^a^, n (%)	55 572 (60%)
Diabetes, n (%)	16 448 (18%)
Hypertension, n (%)	34 822 (38%)
Depression, n (%)	13 565 (15%)
Number of visits over 2 years, mean (SD)	7.9 (7.5)

Physician characteristics	
Physicians, n	180
Sites (office locations), n	46
Mean age, years (SD)	52 (11.2)
Male gender, n (%)	73 (40%)
Number of enrolled patients per practice, n (SD)	611 (597)

Abbreviation: SD, standard deviation.

a Chronic conditions include diabetes, hypertension, or depression.

**Table 2:** Prevalence of 9 chronic disease prevention, screening, and management items in electronic medical records

Category	Description	N (%)
Vitals	Blood pressure measured in past 2 years	75 564 (82%)
	Body mass index measured in past 2 years	56 573 (61%)
	Waist circumference measured in past 2 years	11 348 (12%)
Laboratory	Low‐density lipoprotein measured in past 3 years	70 680 (76%)
	Fasting blood glucose or haemoglobin A1c measured in past 3 years	72 594 (78%)
Lifestyle	Presence of smoking information in the summary health profile	74 124 (80%)
	Presence of alcohol information in the summary health profile	54 260 (59%)
	Presence of diet information in the summary health profile	3320 (4%)
	Presence of exercise information in the summary health profile	14 131 (15%)

There were 282 item sets satisfying the 2% minimum support threshold and 714 rules with at least one CDPSM element in antecedent and a single CDPSM element in consequent. High support was recorded for bivariate rules that predicted the presence of data on BP, smoking, LDL, or A1c. The presence of any CDPSM item in the EMR was associated with confidence greater than 80% that items with high support (BP, A1c, LDL, and smoking) would also be present. Lift ranged from 1.04 to 1.65 and standardized lift ranged from 0.5% to 99.7%. Lift was greater than one for all association rules; this indicated positive correlations among all CDPSM elements. The highest estimates of standardized lift were found for a cluster of association rules predicting the presence of BP.

Lift and standardized lift for the top 10 pairs of CDPSM items (as ranked by standardized lift) are shown in Table 3. Several pairs had high support and high standardized lift; these included [BMI, BP], [A1c, LDL] and [alcohol, smoking].

**Table 3:** Top 10 bivariate rules ranked with respect to standardized lift (rules were generated with minimum support threshold of 2% and minimum confidence threshold of 80%)

Antecedent	Consequent	Support	Confidence	Lift	Standardized Lift
WC	BP	0.12	1.0	1.22	0.99
BMI	BP	0.60	0.98	1.20	0.93
Alcohol	Smoking	0.58	0.98	1.21	0.89
WC	BMI	0.12	0.97	1.59	0.87
Diet	Smoking	0.03	0.97	1.21	0.85
LDL	A1c	0.74	0.97	1.22	0.83
Exercise	Smoking	0.14	0.97	1.20	0.83
A1c	LDL	0.74	0.94	1.22	0.81
Diet	Alcohol	0.03	0.95	1.61	0.75
Exercise	Alcohol	0.14	0.92	1.57	0.63

Abbreviations: A1c, haemoglobin A1c; BMI, body mass index; BP, blood pressure; LDL, low‐density lipoprotein; WC, waist circumference.

We found that 95% (681/714) of the association rules generated from the Apriori algorithm contained redundant information. Using the pruning strategy described by McNicholas et al,^37^ the total number of rules were reduced to 33. These are presented in Table S1. Pruned rules for sets of items predicting the presence of BMI, BP, and alcohol had the highest standardized lift.

**Figure 1:**
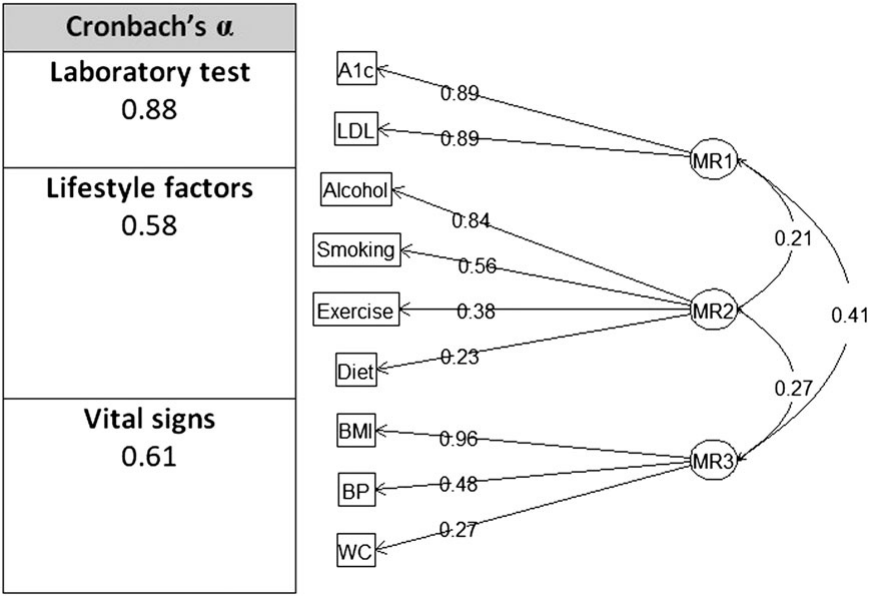
Factor analysis and Cronbach α using oblique rotation of 3 factors (nodes represent principle axis factors and 9 chronic disease prevention, screening, and management elements; edges show tetrachoric correlation). A1c, haemoglobin A1c; BMI, body mass index; BP, blood pressure; LDL, low‐density lipoprotein; WC, waist circumference

Using factor analysis, CDPSM items were grouped into 3 latent categories as shown in Figure 1. Upon inspection, these categories were termed lifestyle factors, vital signs, and laboratory tests. The tetrachoric correlation had high magnitude (>0.5) when the 3 latent categories were compared against their corresponding CDPSM element. The 3 latent categories themselves were also positively correlated with one another. We found a reasonable degree of internal consistency using Cronbach α.

We used association rule mining to examine the provision of CDPSM items across categories as shown in Table 4. The presence of any single item within a category was associated with confidence of more than 80% that at least one item in another category would also be recorded. Lift was greater than one for the 3 categories when using multilevel association rule mining, indicating associations between categories when any CDPSM item was present within a category.

**Table 4:** Multilevel association rules for 3 chronic disease prevention, screening, and management categories

Predictor^a^	Predicted^a^	Support	Confidence	Lift
Laboratory, lifestyle	Vitals	63.2	91.1	1.10
Lifestyle, vitals	Lab	63.2	89.1	1.10
Vitals	Laboratory	72.9	87.9	1.08
Laboratory	Vitals	72.9	89.5	1.08
Laboratory, vitals	Lifestyle	63.2	86.7	1.06
Lifestyle	Laboratory	69.4	84.5	1.04
Lifestyle	Vitals	71.0	86.2	1.04
Laboratory	Lifestyle	69.4	85.3	1.04
Vitals	Lifestyle	71.0	85.6	1.04

a Presence of any single CDPSM item within a category.

**Figure 2:**
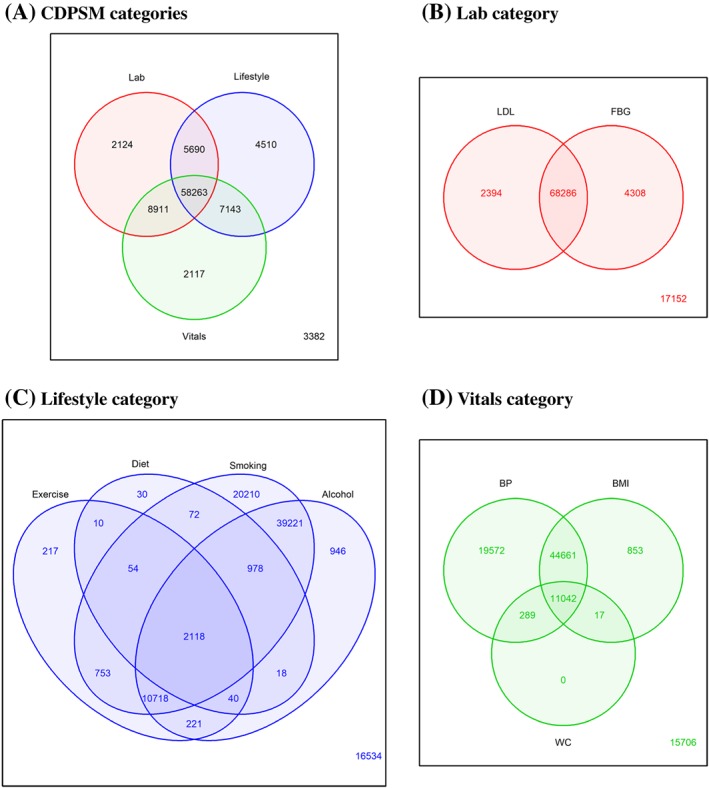
Venn diagrams with number of patients that have chronic disease prevention, screening, and management (CDPSM) items in lab, vitals, and lifestyle categories

**Figure 3:**
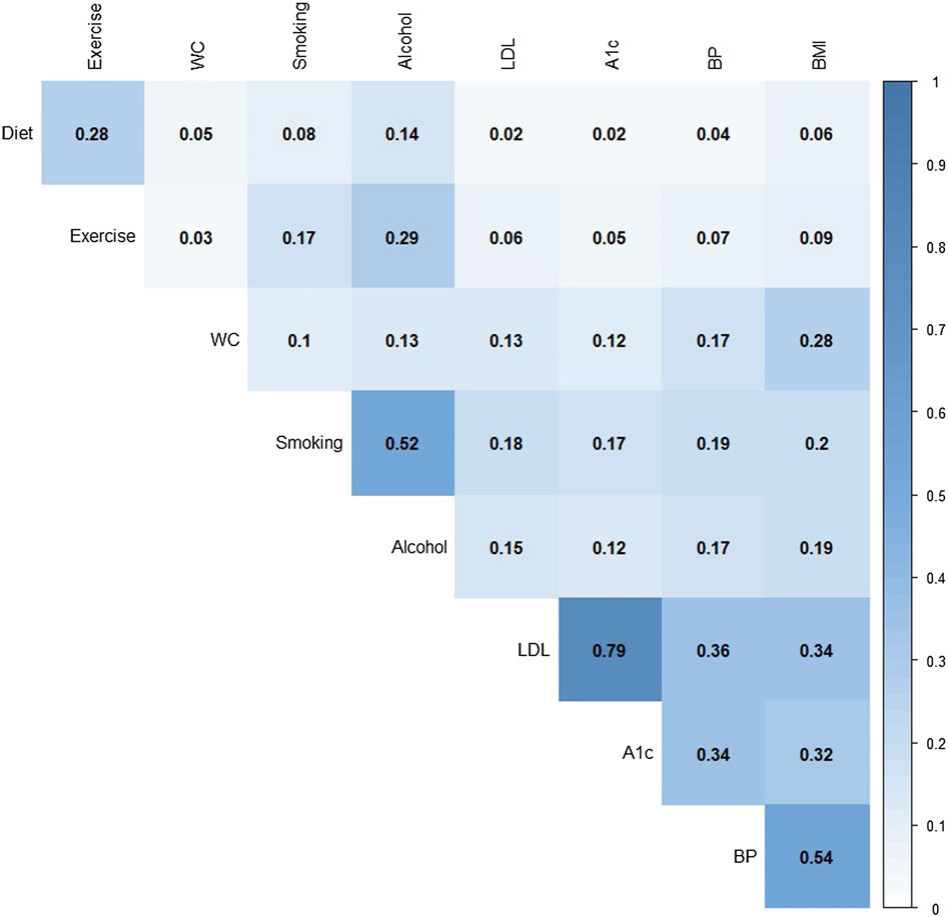
Correlation among 9 chronic disease prevention, screening, and management items. A1c, haemoglobin A1c; BMI, body mass index; BP, blood pressure; LDL, low‐density lipoprotein; WC, waist circumference

Figure 2 shows the occurrence of 9 CDPSM items within each of the 3 categories. Both CDPSM items within the laboratory category were recorded for 68 286 patients (74% of all patients and 91% of patients with lab data present); all 3 CDPSM items within the vital sign category were recorded for 11 042 patients (12% of all patients and 14% of patients with data in vital signs) and all 4 CDPSM items within lifestyle category were recorded for 2118 patients (2.3% of all patients and 2.8% of patients with lifestyle items). Figure 3 shows correlations among 9 CDPSM items. The highest correlation (0.79) was between the 2 laboratory items.

## 4 DISCUSSION

Most patients had at least one screen recorded, but few had all items recorded. In this study, recording any screen was associated with high confidence that BP, lipid levels, glycaemic levels, or smoking status would also be recorded. The presence of any screen within one category was associated over 80% confidence that there would be a screen in another clinical category. Bivariate rules using BMI, alcohol, and laboratory test as predictors had high confidence and standardized lift. All association rules were positively correlated but needed to be considered within the context of other parameters (support and confidence).

A recent systematic review found that computerized decision support systems in EMRs have had somewhat limited effects on morbidity and mortality to date.^41^ Another review found an improvement of about 4% in screening for cardiovascular risk factors associated with clinical decision support.^42^ Decision support systems are usually based on matches between patient characteristics and a computerized knowledge base.^43^ We propose a complementary approach to prompting for screening based on computerized learning of data associations. Using data to influence choices has been highly successful in other domains, such as marketing. Targeting primary care physicians for prompting based on associations may be of benefit as screening choices are strongly influenced by provider decisions and actions.^44^, ^45^ For example, physicians may consciously or unconsciously choose to combine several screens as part of preventive health examinations.^46^

While electronic reminders and prompts based on clinical decision support systems can be effective,^47^, ^48^ “alert fatigue” due to too many prompts or inappropriate reminders may decrease the effectiveness of reminder systems.^25^, ^49^ The use of promising combinations of associated screens may help to refine, calibrate, and focus the system, through the deliberate selection of rules that may be more actionable due to favourable combinations of standardized lift, support, and confidence. As an example, several pairs of items had standardized lift greater than 85%, pointing towards potentially high‐value associations. These may provide an approach to targeted alerting; for example, a contingency‐based EMR alert system could be implemented: “you just recorded a BP, would you also like to record a weight and height?” Peer‐based suggestions derived from association rules, such as “your colleagues also ask about exercise and alcohol use when they record smoking status” may also be effective. Feedback that is immediate and recommends specific activities relevant to the setting and patients may be more likely to lead to clinical action.^50^

A quality improvement activity recommended by the Institute for Health Care Improvement is “max packing” appointments or bundling several appropriate services during a single visit or using fewer visits.^51^ This improves access by reducing the need for future appointments. Clinical prompts derived from associations could be used to increase the number of items recorded in EMRs at each visit.

We have shown that there are associations between the presence of CDPSM items. Further approaches to the study of these associations could consider patient and physician characteristics as well as effects of groups of co‐located physicians.

### 4.1 Strengths and limitations

The study had several strengths. It reflected data from routinely provided primary care for patients. Data were extracted from several different EMR platforms, accounting for a variety of EMR‐specific data entry processes by clinicians. However, there were some important limitations to this study. We recorded items present together in the same chart but not necessarily recorded contemporaneously; for example, smoking status may have been recorded at a different visit than BMI. This was a convenience sample of primary care practices that contributed EMR data to UTOPIAN, rather than a random sample; these physicians may not represent the general population. A recent study of primary care practices contributing data to CPCSSN and its networks have shown that participating physicians are slightly younger and likely to be female compared to the population of physicians who have responded to the National Physician Survey.^27^ In addition, different interestingness measures are not equally good at capturing dependencies among binary attributes and thus the ranking of association rules may vary depending on which interestingness measure is selected.^52^ Nonetheless, the use of this technique provided an efficient method to quantify the relationships among CDPSM items in EMRs.

## 5 CONCLUSIONS

We studied associations among the recording of CDPSM elements in EMRs and ranked important relationships between these elements. This could contribute to planning new approaches for improving the recording of key chronic disease risk factors in primary care through prompts based on associations. Association rule mining and similar approaches appears to be efficient methods to explore relationships between numerous combinations of item sets, as may be encountered in medical transactional databases such as those found in primary care.

## CONFLICT OF INTEREST

The authors declare no conflict of interest.

## AUTHOR CONTRIBUTIONS

SK, MG, BA, and FS contributed to the conception. BA was responsible for acquisition of data. SK, XZ, CM, RM, and BA contributed substantially to the analysis of data. MG drafted the initial version of the article. All authors contributed to the interpretation of data. All authors reviewed and revised the article for important intellectual content and gave final approval of the version to be published.

## Supporting information

Table S1. Details on support, confidence, lift (raw and standardized) for 714 association rules stratified with respect to consequent CDPSM element.
Table S2. Details on support, confidence, lift (raw and standardized) for 33‐pruned association rules stratified with respect to consequent CDPSM element.
