# Supplementary material for: Would you like to add a weight after this blood pressure, doctor? Discovery of potentially actionable associations between the provision of multiple screens in primary care
Source: J Eval Clin Pract. 2018 Jan 19;24(2):423–30. doi: 10.1111/jep.12877 (PMC5900937; doi:10.1111/jep.12877)
Supplement: Supplementary file 1 — Table S1. Details on support, confidence, lift (raw and standardized) for 714 association rules stratified with respect to consequent CDPSM element. Table S2. Details on support, confidence, lift (raw and standardized) for 33‐pruned association rules stratified with respect to consequent CDPSM element. [file JEP-24-423-s001.docx]

# Supporting information

**S1 Table. Details on support, confidence, lift (raw and standardized) for 714 association rules stratified with respect to consequent CDPSM element.**

| Rule number | Antecedent | Consequent | Support | Confidence | Lift | Support for Antecedent | Support for Consequent | Standardized lift |
| --- | --- | --- | --- | --- | --- | --- | --- | --- |
| 1 | {WC_2Y,LDL_3Y,FBG_A1c_3Y,Exercise} | {BP_2Y} | 0.020 | 0.999 | 1.219 | 0.020 | 0.820 | 0.545 |
| 2 | {BMI_2Y,WC_2Y,FBG_A1c_3Y,Exercise} | {BP_2Y} | 0.020 | 0.999 | 1.219 | 0.020 | 0.820 | 0.861 |
| 3 | {WC_2Y,LDL_3Y,Exercise} | {BP_2Y} | 0.020 | 0.999 | 1.219 | 0.020 | 0.820 | 0.953 |
| 4 | {WC_2Y,FBG_A1c_3Y,Exercise} | {BP_2Y} | 0.020 | 0.999 | 1.219 | 0.020 | 0.820 | 0.968 |
| 5 | {Smoking,Alcohol,Diet,Exercise} | {BP_2Y} | 0.021 | 0.896 | 1.093 | 0.023 | 0.820 | 0.201 |
| 6 | {Alcohol,Diet,Exercise} | {BP_2Y} | 0.021 | 0.895 | 1.092 | 0.023 | 0.820 | 0.283 |
| 7 | {Smoking,Diet,Exercise} | {BP_2Y} | 0.021 | 0.896 | 1.092 | 0.024 | 0.820 | 0.313 |
| 8 | {BMI_2Y,WC_2Y,Smoking,Exercise} | {BP_2Y} | 0.021 | 0.999 | 1.218 | 0.021 | 0.820 | 0.983 |
| 9 | {WC_2Y,Smoking,Exercise} | {BP_2Y} | 0.022 | 0.999 | 1.218 | 0.022 | 0.820 | 0.986 |
| 10 | {Diet,Exercise} | {BP_2Y} | 0.022 | 0.895 | 1.092 | 0.024 | 0.820 | 0.386 |
| 11 | {BMI_2Y,WC_2Y,Exercise} | {BP_2Y} | 0.022 | 0.999 | 1.218 | 0.022 | 0.820 | 0.989 |
| 12 | {WC_2Y,Exercise} | {BP_2Y} | 0.022 | 0.999 | 1.218 | 0.022 | 0.820 | 0.990 |
| 13 | {BMI_2Y,LDL_3Y,FBG_A1c_3Y,Smoking,Alcohol,Diet} | {BP_2Y} | 0.023 | 0.994 | 1.213 | 0.023 | 0.820 | 0.959 |
| 14 | {BMI_2Y,LDL_3Y,Smoking,Alcohol,Diet} | {BP_2Y} | 0.023 | 0.994 | 1.212 | 0.023 | 0.820 | 0.959 |
| 15 | {BMI_2Y,LDL_3Y,FBG_A1c_3Y,Alcohol,Diet} | {BP_2Y} | 0.023 | 0.994 | 1.212 | 0.023 | 0.820 | 0.959 |
| 16 | {BMI_2Y,LDL_3Y,Alcohol,Diet} | {BP_2Y} | 0.024 | 0.994 | 1.212 | 0.024 | 0.820 | 0.959 |
| 17 | {BMI_2Y,FBG_A1c_3Y,Smoking,Alcohol,Diet} | {BP_2Y} | 0.024 | 0.994 | 1.212 | 0.024 | 0.820 | 0.962 |
| 18 | {BMI_2Y,LDL_3Y,FBG_A1c_3Y,Smoking,Diet} | {BP_2Y} | 0.024 | 0.995 | 1.213 | 0.024 | 0.820 | 0.968 |
| 19 | {BMI_2Y,FBG_A1c_3Y,Alcohol,Diet} | {BP_2Y} | 0.024 | 0.993 | 1.211 | 0.024 | 0.820 | 0.962 |
| 20 | {BMI_2Y,LDL_3Y,Smoking,Diet} | {BP_2Y} | 0.024 | 0.994 | 1.212 | 0.024 | 0.820 | 0.967 |
| 21 | {BMI_2Y,LDL_3Y,FBG_A1c_3Y,Diet} | {BP_2Y} | 0.024 | 0.994 | 1.212 | 0.025 | 0.820 | 0.967 |
| 22 | {BMI_2Y,LDL_3Y,Diet} | {BP_2Y} | 0.025 | 0.993 | 1.211 | 0.025 | 0.820 | 0.967 |
| 23 | {BMI_2Y,FBG_A1c_3Y,Smoking,Diet} | {BP_2Y} | 0.025 | 0.994 | 1.212 | 0.025 | 0.820 | 0.969 |
| 24 | {BMI_2Y,FBG_A1c_3Y,Diet} | {BP_2Y} | 0.025 | 0.993 | 1.211 | 0.025 | 0.820 | 0.966 |
| 25 | {LDL_3Y,FBG_A1c_3Y,Smoking,Alcohol,Diet} | {BP_2Y} | 0.026 | 0.953 | 1.162 | 0.027 | 0.820 | 0.765 |
| 26 | {BMI_2Y,Smoking,Alcohol,Diet} | {BP_2Y} | 0.026 | 0.993 | 1.210 | 0.026 | 0.820 | 0.963 |
| 27 | {LDL_3Y,Smoking,Alcohol,Diet} | {BP_2Y} | 0.026 | 0.952 | 1.161 | 0.027 | 0.820 | 0.761 |
| 28 | {LDL_3Y,FBG_A1c_3Y,Alcohol,Diet} | {BP_2Y} | 0.026 | 0.953 | 1.162 | 0.027 | 0.820 | 0.765 |
| 29 | {BMI_2Y,Alcohol,Diet} | {BP_2Y} | 0.026 | 0.992 | 1.209 | 0.027 | 0.820 | 0.959 |
| 30 | {LDL_3Y,Alcohol,Diet} | {BP_2Y} | 0.027 | 0.952 | 1.161 | 0.028 | 0.820 | 0.761 |
| 31 | {FBG_A1c_3Y,Smoking,Alcohol,Diet} | {BP_2Y} | 0.027 | 0.948 | 1.156 | 0.028 | 0.820 | 0.738 |
| 32 | {LDL_3Y,FBG_A1c_3Y,Smoking,Diet} | {BP_2Y} | 0.027 | 0.953 | 1.163 | 0.028 | 0.820 | 0.767 |
| 33 | {BMI_2Y,Smoking,Diet} | {BP_2Y} | 0.027 | 0.992 | 1.210 | 0.027 | 0.820 | 0.962 |
| 34 | {FBG_A1c_3Y,Alcohol,Diet} | {BP_2Y} | 0.027 | 0.947 | 1.155 | 0.029 | 0.820 | 0.737 |
| 35 | {LDL_3Y,Smoking,Diet} | {BP_2Y} | 0.027 | 0.953 | 1.161 | 0.029 | 0.820 | 0.763 |
| 36 | {LDL_3Y,FBG_A1c_3Y,Diet} | {BP_2Y} | 0.028 | 0.953 | 1.162 | 0.029 | 0.820 | 0.763 |
| 37 | {BMI_2Y,Diet} | {BP_2Y} | 0.028 | 0.991 | 1.209 | 0.028 | 0.820 | 0.957 |
| 38 | {FBG_A1c_3Y,Smoking,Diet} | {BP_2Y} | 0.028 | 0.948 | 1.156 | 0.029 | 0.820 | 0.741 |
| 39 | {LDL_3Y,Diet} | {BP_2Y} | 0.028 | 0.952 | 1.161 | 0.029 | 0.820 | 0.760 |
| 40 | {FBG_A1c_3Y,Diet} | {BP_2Y} | 0.029 | 0.947 | 1.155 | 0.030 | 0.820 | 0.735 |
| 41 | {Smoking,Alcohol,Diet} | {BP_2Y} | 0.030 | 0.891 | 1.086 | 0.034 | 0.820 | 0.454 |
| 42 | {Alcohol,Diet} | {BP_2Y} | 0.030 | 0.890 | 1.085 | 0.034 | 0.820 | 0.450 |
| 43 | {Smoking,Diet} | {BP_2Y} | 0.031 | 0.891 | 1.087 | 0.035 | 0.820 | 0.457 |
| 44 | {Diet} | {BP_2Y} | 0.032 | 0.890 | 1.086 | 0.036 | 0.820 | 0.452 |
| 45 | {BMI_2Y,WC_2Y,LDL_3Y,FBG_A1c_3Y,Smoking,Alcohol} | {BP_2Y} | 0.080 | 0.999 | 1.219 | 0.080 | 0.820 | 0.997 |
| 46 | {BMI_2Y,WC_2Y,LDL_3Y,FBG_A1c_3Y,Alcohol} | {BP_2Y} | 0.082 | 0.999 | 1.219 | 0.082 | 0.820 | 0.997 |
| 47 | {WC_2Y,LDL_3Y,FBG_A1c_3Y,Smoking,Alcohol} | {BP_2Y} | 0.082 | 0.999 | 1.219 | 0.082 | 0.820 | 0.997 |
| 48 | {BMI_2Y,WC_2Y,LDL_3Y,Smoking,Alcohol} | {BP_2Y} | 0.082 | 0.999 | 1.218 | 0.082 | 0.820 | 0.996 |
| 49 | {BMI_2Y,WC_2Y,FBG_A1c_3Y,Smoking,Alcohol} | {BP_2Y} | 0.082 | 0.999 | 1.219 | 0.082 | 0.820 | 0.997 |
| 50 | {WC_2Y,LDL_3Y,FBG_A1c_3Y,Alcohol} | {BP_2Y} | 0.083 | 0.999 | 1.219 | 0.083 | 0.820 | 0.997 |
| 51 | {BMI_2Y,WC_2Y,LDL_3Y,Alcohol} | {BP_2Y} | 0.083 | 0.999 | 1.218 | 0.083 | 0.820 | 0.995 |
| 52 | {WC_2Y,LDL_3Y,Smoking,Alcohol} | {BP_2Y} | 0.083 | 0.999 | 1.218 | 0.083 | 0.820 | 0.996 |
| 53 | {BMI_2Y,WC_2Y,FBG_A1c_3Y,Alcohol} | {BP_2Y} | 0.084 | 0.999 | 1.218 | 0.084 | 0.820 | 0.996 |
| 54 | {WC_2Y,FBG_A1c_3Y,Smoking,Alcohol} | {BP_2Y} | 0.084 | 0.999 | 1.219 | 0.084 | 0.820 | 0.997 |
| 55 | {WC_2Y,LDL_3Y,Alcohol} | {BP_2Y} | 0.085 | 0.999 | 1.218 | 0.085 | 0.820 | 0.996 |
| 56 | {WC_2Y,FBG_A1c_3Y,Alcohol} | {BP_2Y} | 0.085 | 0.999 | 1.218 | 0.085 | 0.820 | 0.996 |
| 57 | {BMI_2Y,LDL_3Y,FBG_A1c_3Y,Smoking,Alcohol,Exercise} | {BP_2Y} | 0.089 | 0.992 | 1.209 | 0.090 | 0.820 | 0.959 |
| 58 | {BMI_2Y,LDL_3Y,FBG_A1c_3Y,Alcohol,Exercise} | {BP_2Y} | 0.090 | 0.992 | 1.209 | 0.091 | 0.820 | 0.958 |
| 59 | {BMI_2Y,WC_2Y,Smoking,Alcohol} | {BP_2Y} | 0.091 | 0.999 | 1.218 | 0.091 | 0.820 | 0.996 |
| 60 | {BMI_2Y,LDL_3Y,Smoking,Alcohol,Exercise} | {BP_2Y} | 0.091 | 0.992 | 1.209 | 0.092 | 0.820 | 0.959 |
| 61 | {BMI_2Y,FBG_A1c_3Y,Smoking,Alcohol,Exercise} | {BP_2Y} | 0.091 | 0.992 | 1.209 | 0.092 | 0.820 | 0.958 |
| 62 | {WC_2Y,Smoking,Alcohol} | {BP_2Y} | 0.092 | 0.999 | 1.218 | 0.092 | 0.820 | 0.996 |
| 63 | {BMI_2Y,WC_2Y,Alcohol} | {BP_2Y} | 0.092 | 0.999 | 1.218 | 0.092 | 0.820 | 0.995 |
| 64 | {BMI_2Y,LDL_3Y,Alcohol,Exercise} | {BP_2Y} | 0.093 | 0.992 | 1.209 | 0.093 | 0.820 | 0.958 |
| 65 | {BMI_2Y,FBG_A1c_3Y,Alcohol,Exercise} | {BP_2Y} | 0.093 | 0.991 | 1.209 | 0.094 | 0.820 | 0.957 |
| 66 | {WC_2Y,Alcohol} | {BP_2Y} | 0.093 | 0.999 | 1.218 | 0.093 | 0.820 | 0.995 |
| 67 | {BMI_2Y,LDL_3Y,FBG_A1c_3Y,Smoking,Exercise} | {BP_2Y} | 0.094 | 0.992 | 1.209 | 0.095 | 0.820 | 0.959 |
| 68 | {BMI_2Y,LDL_3Y,Smoking,Exercise} | {BP_2Y} | 0.096 | 0.992 | 1.209 | 0.097 | 0.820 | 0.959 |
| 69 | {BMI_2Y,FBG_A1c_3Y,Smoking,Exercise} | {BP_2Y} | 0.096 | 0.992 | 1.209 | 0.097 | 0.820 | 0.958 |
| 70 | {BMI_2Y,LDL_3Y,FBG_A1c_3Y,Exercise} | {BP_2Y} | 0.096 | 0.992 | 1.209 | 0.097 | 0.820 | 0.959 |
| 71 | {BMI_2Y,WC_2Y,LDL_3Y,FBG_A1c_3Y,Smoking} | {BP_2Y} | 0.098 | 0.999 | 1.218 | 0.098 | 0.820 | 0.996 |
| 72 | {BMI_2Y,LDL_3Y,Exercise} | {BP_2Y} | 0.099 | 0.992 | 1.209 | 0.100 | 0.820 | 0.959 |
| 73 | {BMI_2Y,FBG_A1c_3Y,Exercise} | {BP_2Y} | 0.099 | 0.992 | 1.209 | 0.100 | 0.820 | 0.958 |
| 74 | {BMI_2Y,WC_2Y,LDL_3Y,Smoking} | {BP_2Y} | 0.100 | 0.999 | 1.218 | 0.100 | 0.820 | 0.995 |
| 75 | {WC_2Y,LDL_3Y,FBG_A1c_3Y,Smoking} | {BP_2Y} | 0.100 | 0.999 | 1.218 | 0.100 | 0.820 | 0.996 |
| 76 | {BMI_2Y,WC_2Y,FBG_A1c_3Y,Smoking} | {BP_2Y} | 0.100 | 0.999 | 1.218 | 0.101 | 0.820 | 0.996 |
| 77 | {BMI_2Y,Smoking,Alcohol,Exercise} | {BP_2Y} | 0.101 | 0.990 | 1.208 | 0.102 | 0.820 | 0.951 |
| 78 | {BMI_2Y,Alcohol,Exercise} | {BP_2Y} | 0.102 | 0.990 | 1.207 | 0.103 | 0.820 | 0.951 |
| 79 | {WC_2Y,LDL_3Y,Smoking} | {BP_2Y} | 0.102 | 0.999 | 1.218 | 0.103 | 0.820 | 0.995 |
| 80 | {WC_2Y,FBG_A1c_3Y,Smoking} | {BP_2Y} | 0.103 | 0.999 | 1.218 | 0.103 | 0.820 | 0.996 |
| 81 | {LDL_3Y,FBG_A1c_3Y,Smoking,Alcohol,Exercise} | {BP_2Y} | 0.106 | 0.931 | 1.135 | 0.113 | 0.820 | 0.654 |
| 82 | {BMI_2Y,Smoking,Exercise} | {BP_2Y} | 0.106 | 0.990 | 1.208 | 0.107 | 0.820 | 0.951 |
| 83 | {BMI_2Y,WC_2Y,LDL_3Y,FBG_A1c_3Y} | {BP_2Y} | 0.107 | 0.999 | 1.218 | 0.107 | 0.820 | 0.994 |
| 84 | {LDL_3Y,FBG_A1c_3Y,Alcohol,Exercise} | {BP_2Y} | 0.107 | 0.930 | 1.135 | 0.115 | 0.820 | 0.652 |
| 85 | {LDL_3Y,Smoking,Alcohol,Exercise} | {BP_2Y} | 0.108 | 0.930 | 1.133 | 0.117 | 0.820 | 0.648 |
| 86 | {FBG_A1c_3Y,Smoking,Alcohol,Exercise} | {BP_2Y} | 0.109 | 0.928 | 1.131 | 0.117 | 0.820 | 0.639 |
| 87 | {BMI_2Y,WC_2Y,LDL_3Y} | {BP_2Y} | 0.109 | 0.999 | 1.218 | 0.110 | 0.820 | 0.994 |
| 88 | {BMI_2Y,WC_2Y,Smoking} | {BP_2Y} | 0.109 | 0.999 | 1.218 | 0.110 | 0.820 | 0.995 |
| 89 | {BMI_2Y,Exercise} | {BP_2Y} | 0.110 | 0.990 | 1.207 | 0.111 | 0.820 | 0.951 |
| 90 | {BMI_2Y,WC_2Y,FBG_A1c_3Y} | {BP_2Y} | 0.110 | 0.999 | 1.218 | 0.110 | 0.820 | 0.994 |
| 91 | {WC_2Y,LDL_3Y,FBG_A1c_3Y} | {BP_2Y} | 0.110 | 0.999 | 1.218 | 0.110 | 0.820 | 0.995 |
| 92 | {LDL_3Y,Alcohol,Exercise} | {BP_2Y} | 0.110 | 0.929 | 1.133 | 0.119 | 0.820 | 0.647 |
| 93 | {FBG_A1c_3Y,Alcohol,Exercise} | {BP_2Y} | 0.111 | 0.927 | 1.131 | 0.120 | 0.820 | 0.637 |
| 94 | {LDL_3Y,FBG_A1c_3Y,Smoking,Exercise} | {BP_2Y} | 0.112 | 0.932 | 1.136 | 0.120 | 0.820 | 0.658 |
| 95 | {WC_2Y,Smoking} | {BP_2Y} | 0.112 | 0.999 | 1.218 | 0.112 | 0.820 | 0.995 |
| 96 | {WC_2Y,LDL_3Y} | {BP_2Y} | 0.112 | 0.999 | 1.218 | 0.112 | 0.820 | 0.994 |
| 97 | {WC_2Y,FBG_A1c_3Y} | {BP_2Y} | 0.113 | 0.999 | 1.218 | 0.113 | 0.820 | 0.994 |
| 98 | {LDL_3Y,Smoking,Exercise} | {BP_2Y} | 0.115 | 0.930 | 1.135 | 0.124 | 0.820 | 0.652 |
| 99 | {LDL_3Y,FBG_A1c_3Y,Exercise} | {BP_2Y} | 0.115 | 0.931 | 1.136 | 0.124 | 0.820 | 0.657 |
| 100 | {FBG_A1c_3Y,Smoking,Exercise} | {BP_2Y} | 0.116 | 0.929 | 1.132 | 0.125 | 0.820 | 0.643 |
| 101 | {LDL_3Y,Exercise} | {BP_2Y} | 0.118 | 0.930 | 1.134 | 0.127 | 0.820 | 0.650 |
| 102 | {FBG_A1c_3Y,Exercise} | {BP_2Y} | 0.119 | 0.928 | 1.132 | 0.129 | 0.820 | 0.641 |
| 103 | {BMI_2Y,WC_2Y} | {BP_2Y} | 0.120 | 0.998 | 1.217 | 0.120 | 0.820 | 0.992 |
| 104 | {WC_2Y} | {BP_2Y} | 0.123 | 0.999 | 1.218 | 0.123 | 0.820 | 0.993 |
| 105 | {Smoking,Alcohol,Exercise} | {BP_2Y} | 0.123 | 0.886 | 1.080 | 0.139 | 0.820 | 0.428 |
| 106 | {Alcohol,Exercise} | {BP_2Y} | 0.126 | 0.885 | 1.079 | 0.142 | 0.820 | 0.425 |
| 107 | {Smoking,Exercise} | {BP_2Y} | 0.131 | 0.886 | 1.081 | 0.148 | 0.820 | 0.432 |
| 108 | {Exercise} | {BP_2Y} | 0.136 | 0.886 | 1.080 | 0.153 | 0.820 | 0.429 |
| 109 | {BMI_2Y,LDL_3Y,FBG_A1c_3Y,Smoking,Alcohol} | {BP_2Y} | 0.345 | 0.989 | 1.206 | 0.348 | 0.820 | 0.945 |
| 110 | {BMI_2Y,LDL_3Y,FBG_A1c_3Y,Alcohol} | {BP_2Y} | 0.351 | 0.989 | 1.206 | 0.355 | 0.820 | 0.945 |
| 111 | {BMI_2Y,LDL_3Y,Smoking,Alcohol} | {BP_2Y} | 0.353 | 0.989 | 1.206 | 0.357 | 0.820 | 0.945 |
| 112 | {BMI_2Y,FBG_A1c_3Y,Smoking,Alcohol} | {BP_2Y} | 0.355 | 0.989 | 1.205 | 0.360 | 0.820 | 0.943 |
| 113 | {BMI_2Y,LDL_3Y,Alcohol} | {BP_2Y} | 0.360 | 0.989 | 1.206 | 0.364 | 0.820 | 0.945 |
| 114 | {BMI_2Y,FBG_A1c_3Y,Alcohol} | {BP_2Y} | 0.362 | 0.989 | 1.205 | 0.366 | 0.820 | 0.943 |
| 115 | {BMI_2Y,Smoking,Alcohol} | {BP_2Y} | 0.394 | 0.987 | 1.204 | 0.399 | 0.820 | 0.935 |
| 116 | {BMI_2Y,Alcohol} | {BP_2Y} | 0.401 | 0.987 | 1.204 | 0.407 | 0.820 | 0.935 |
| 117 | {LDL_3Y,FBG_A1c_3Y,Smoking,Alcohol} | {BP_2Y} | 0.424 | 0.926 | 1.129 | 0.458 | 0.820 | 0.628 |
| 118 | {LDL_3Y,FBG_A1c_3Y,Alcohol} | {BP_2Y} | 0.432 | 0.925 | 1.128 | 0.467 | 0.820 | 0.626 |
| 119 | {LDL_3Y,Smoking,Alcohol} | {BP_2Y} | 0.437 | 0.924 | 1.127 | 0.473 | 0.820 | 0.621 |
| 120 | {FBG_A1c_3Y,Smoking,Alcohol} | {BP_2Y} | 0.441 | 0.921 | 1.123 | 0.479 | 0.820 | 0.607 |
| 121 | {LDL_3Y,Alcohol} | {BP_2Y} | 0.445 | 0.924 | 1.126 | 0.482 | 0.820 | 0.618 |
| 122 | {FBG_A1c_3Y,Alcohol} | {BP_2Y} | 0.450 | 0.921 | 1.123 | 0.489 | 0.820 | 0.604 |
| 123 | {BMI_2Y,LDL_3Y,FBG_A1c_3Y,Smoking} | {BP_2Y} | 0.456 | 0.988 | 1.205 | 0.461 | 0.820 | 0.942 |
| 124 | {BMI_2Y,LDL_3Y,Smoking} | {BP_2Y} | 0.467 | 0.988 | 1.205 | 0.473 | 0.820 | 0.941 |
| 125 | {BMI_2Y,FBG_A1c_3Y,Smoking} | {BP_2Y} | 0.471 | 0.988 | 1.204 | 0.477 | 0.820 | 0.938 |
| 126 | {Smoking,Alcohol} | {BP_2Y} | 0.505 | 0.877 | 1.069 | 0.576 | 0.820 | 0.384 |
| 127 | {Alcohol} | {BP_2Y} | 0.516 | 0.875 | 1.068 | 0.589 | 0.820 | 0.377 |
| 128 | {BMI_2Y,LDL_3Y,FBG_A1c_3Y} | {BP_2Y} | 0.520 | 0.988 | 1.204 | 0.526 | 0.820 | 0.939 |
| 129 | {BMI_2Y,Smoking} | {BP_2Y} | 0.524 | 0.986 | 1.202 | 0.532 | 0.820 | 0.928 |
| 130 | {BMI_2Y,LDL_3Y} | {BP_2Y} | 0.535 | 0.988 | 1.204 | 0.541 | 0.820 | 0.938 |
| 131 | {BMI_2Y,FBG_A1c_3Y} | {BP_2Y} | 0.540 | 0.987 | 1.203 | 0.547 | 0.820 | 0.935 |
| 132 | {LDL_3Y,FBG_A1c_3Y,Smoking} | {BP_2Y} | 0.572 | 0.912 | 1.112 | 0.628 | 0.820 | 0.559 |
| 133 | {LDL_3Y,Smoking} | {BP_2Y} | 0.589 | 0.910 | 1.110 | 0.648 | 0.820 | 0.552 |
| 134 | {FBG_A1c_3Y,Smoking} | {BP_2Y} | 0.598 | 0.903 | 1.102 | 0.662 | 0.820 | 0.517 |
| 135 | {BMI_2Y} | {BP_2Y} | 0.605 | 0.985 | 1.201 | 0.614 | 0.820 | 0.923 |
| 136 | {LDL_3Y,FBG_A1c_3Y} | {BP_2Y} | 0.666 | 0.898 | 1.095 | 0.741 | 0.820 | 0.491 |
| 137 | {LDL_3Y} | {BP_2Y} | 0.687 | 0.896 | 1.093 | 0.767 | 0.820 | 0.481 |
| 138 | {Smoking} | {BP_2Y} | 0.689 | 0.856 | 1.044 | 0.804 | 0.820 | 0.280 |
| 139 | {FBG_A1c_3Y} | {BP_2Y} | 0.700 | 0.888 | 1.083 | 0.788 | 0.820 | 0.442 |
| 140 | {BP_2Y,WC_2Y,FBG_A1c_3Y,Exercise} | {LDL_3Y} | 0.020 | 0.985 | 1.283 | 0.020 | 0.767 | 0.040 |
| 141 | {Diet,Exercise} | {LDL_3Y} | 0.020 | 0.830 | 1.082 | 0.024 | 0.767 | 0.006 |
| 142 | {WC_2Y,FBG_A1c_3Y,Exercise} | {LDL_3Y} | 0.020 | 0.985 | 1.283 | 0.020 | 0.767 | 0.071 |
| 143 | {BP_2Y,WC_2Y,Exercise} | {LDL_3Y} | 0.020 | 0.910 | 1.186 | 0.022 | 0.767 | 0.098 |
| 144 | {WC_2Y,Exercise} | {LDL_3Y} | 0.020 | 0.909 | 1.185 | 0.022 | 0.767 | 0.102 |
| 145 | {BP_2Y,BMI_2Y,FBG_A1c_3Y,Smoking,Alcohol,Diet} | {LDL_3Y} | 0.023 | 0.967 | 1.261 | 0.024 | 0.767 | 0.794 |
| 146 | {BMI_2Y,FBG_A1c_3Y,Smoking,Alcohol,Diet} | {LDL_3Y} | 0.023 | 0.966 | 1.260 | 0.024 | 0.767 | 0.797 |
| 147 | {BP_2Y,BMI_2Y,Smoking,Alcohol,Diet} | {LDL_3Y} | 0.023 | 0.898 | 1.170 | 0.026 | 0.767 | 0.489 |
| 148 | {BP_2Y,BMI_2Y,FBG_A1c_3Y,Alcohol,Diet} | {LDL_3Y} | 0.023 | 0.966 | 1.260 | 0.024 | 0.767 | 0.803 |
| 149 | {BMI_2Y,Smoking,Alcohol,Diet} | {LDL_3Y} | 0.023 | 0.897 | 1.169 | 0.026 | 0.767 | 0.483 |
| 150 | {BMI_2Y,FBG_A1c_3Y,Alcohol,Diet} | {LDL_3Y} | 0.023 | 0.966 | 1.259 | 0.024 | 0.767 | 0.806 |
| 151 | {BP_2Y,BMI_2Y,Alcohol,Diet} | {LDL_3Y} | 0.024 | 0.896 | 1.168 | 0.026 | 0.767 | 0.479 |
| 152 | {BMI_2Y,Alcohol,Diet} | {LDL_3Y} | 0.024 | 0.894 | 1.166 | 0.027 | 0.767 | 0.471 |
| 153 | {BP_2Y,BMI_2Y,FBG_A1c_3Y,Smoking,Diet} | {LDL_3Y} | 0.024 | 0.968 | 1.261 | 0.025 | 0.767 | 0.830 |
| 154 | {BMI_2Y,FBG_A1c_3Y,Smoking,Diet} | {LDL_3Y} | 0.024 | 0.967 | 1.260 | 0.025 | 0.767 | 0.831 |
| 155 | {BP_2Y,BMI_2Y,Smoking,Diet} | {LDL_3Y} | 0.024 | 0.898 | 1.171 | 0.027 | 0.767 | 0.492 |
| 156 | {BMI_2Y,Smoking,Diet} | {LDL_3Y} | 0.024 | 0.897 | 1.169 | 0.027 | 0.767 | 0.484 |
| 157 | {BP_2Y,BMI_2Y,FBG_A1c_3Y,Diet} | {LDL_3Y} | 0.024 | 0.967 | 1.261 | 0.025 | 0.767 | 0.835 |
| 158 | {BMI_2Y,FBG_A1c_3Y,Diet} | {LDL_3Y} | 0.025 | 0.966 | 1.260 | 0.025 | 0.767 | 0.831 |
| 159 | {BP_2Y,BMI_2Y,Diet} | {LDL_3Y} | 0.025 | 0.897 | 1.169 | 0.028 | 0.767 | 0.484 |
| 160 | {BMI_2Y,Diet} | {LDL_3Y} | 0.025 | 0.895 | 1.167 | 0.028 | 0.767 | 0.475 |
| 161 | {BP_2Y,FBG_A1c_3Y,Smoking,Alcohol,Diet} | {LDL_3Y} | 0.026 | 0.965 | 1.258 | 0.027 | 0.767 | 0.823 |
| 162 | {BP_2Y,Smoking,Alcohol,Diet} | {LDL_3Y} | 0.026 | 0.874 | 1.139 | 0.030 | 0.767 | 0.369 |
| 163 | {BP_2Y,FBG_A1c_3Y,Alcohol,Diet} | {LDL_3Y} | 0.026 | 0.964 | 1.257 | 0.027 | 0.767 | 0.820 |
| 164 | {BP_2Y,Alcohol,Diet} | {LDL_3Y} | 0.027 | 0.871 | 1.136 | 0.030 | 0.767 | 0.355 |
| 165 | {BP_2Y,FBG_A1c_3Y,Smoking,Diet} | {LDL_3Y} | 0.027 | 0.965 | 1.258 | 0.028 | 0.767 | 0.825 |
| 166 | {FBG_A1c_3Y,Smoking,Alcohol,Diet} | {LDL_3Y} | 0.027 | 0.959 | 1.250 | 0.028 | 0.767 | 0.796 |
| 167 | {BP_2Y,Smoking,Diet} | {LDL_3Y} | 0.027 | 0.874 | 1.139 | 0.031 | 0.767 | 0.368 |
| 168 | {FBG_A1c_3Y,Alcohol,Diet} | {LDL_3Y} | 0.027 | 0.958 | 1.249 | 0.029 | 0.767 | 0.792 |
| 169 | {Smoking,Alcohol,Diet} | {LDL_3Y} | 0.027 | 0.818 | 1.066 | 0.034 | 0.767 | 0.088 |
| 170 | {BP_2Y,FBG_A1c_3Y,Diet} | {LDL_3Y} | 0.028 | 0.964 | 1.257 | 0.029 | 0.767 | 0.821 |
| 171 | {Alcohol,Diet} | {LDL_3Y} | 0.028 | 0.814 | 1.061 | 0.034 | 0.767 | 0.071 |
| 172 | {BP_2Y,Diet} | {LDL_3Y} | 0.028 | 0.871 | 1.136 | 0.032 | 0.767 | 0.356 |
| 173 | {FBG_A1c_3Y,Smoking,Diet} | {LDL_3Y} | 0.028 | 0.960 | 1.251 | 0.029 | 0.767 | 0.799 |
| 174 | {Smoking,Diet} | {LDL_3Y} | 0.029 | 0.818 | 1.066 | 0.035 | 0.767 | 0.088 |
| 175 | {FBG_A1c_3Y,Diet} | {LDL_3Y} | 0.029 | 0.959 | 1.250 | 0.030 | 0.767 | 0.793 |
| 176 | {Diet} | {LDL_3Y} | 0.029 | 0.815 | 1.062 | 0.036 | 0.767 | 0.074 |
| 177 | {BP_2Y,BMI_2Y,WC_2Y,FBG_A1c_3Y,Smoking,Alcohol} | {LDL_3Y} | 0.080 | 0.976 | 1.272 | 0.082 | 0.767 | 0.878 |
| 178 | {BMI_2Y,WC_2Y,FBG_A1c_3Y,Smoking,Alcohol} | {LDL_3Y} | 0.080 | 0.976 | 1.272 | 0.082 | 0.767 | 0.878 |
| 179 | {BP_2Y,BMI_2Y,WC_2Y,FBG_A1c_3Y,Alcohol} | {LDL_3Y} | 0.082 | 0.976 | 1.272 | 0.084 | 0.767 | 0.878 |
| 180 | {BP_2Y,WC_2Y,FBG_A1c_3Y,Smoking,Alcohol} | {LDL_3Y} | 0.082 | 0.976 | 1.272 | 0.084 | 0.767 | 0.878 |
| 181 | {BMI_2Y,WC_2Y,FBG_A1c_3Y,Alcohol} | {LDL_3Y} | 0.082 | 0.975 | 1.272 | 0.084 | 0.767 | 0.877 |
| 182 | {WC_2Y,FBG_A1c_3Y,Smoking,Alcohol} | {LDL_3Y} | 0.082 | 0.975 | 1.272 | 0.084 | 0.767 | 0.877 |
| 183 | {BP_2Y,BMI_2Y,WC_2Y,Smoking,Alcohol} | {LDL_3Y} | 0.082 | 0.909 | 1.185 | 0.091 | 0.767 | 0.546 |
| 184 | {BMI_2Y,WC_2Y,Smoking,Alcohol} | {LDL_3Y} | 0.082 | 0.909 | 1.185 | 0.091 | 0.767 | 0.546 |
| 185 | {BP_2Y,WC_2Y,FBG_A1c_3Y,Alcohol} | {LDL_3Y} | 0.083 | 0.976 | 1.272 | 0.085 | 0.767 | 0.878 |
| 186 | {WC_2Y,FBG_A1c_3Y,Alcohol} | {LDL_3Y} | 0.083 | 0.975 | 1.272 | 0.085 | 0.767 | 0.877 |
| 187 | {BP_2Y,BMI_2Y,WC_2Y,Alcohol} | {LDL_3Y} | 0.083 | 0.909 | 1.184 | 0.092 | 0.767 | 0.543 |
| 188 | {BP_2Y,WC_2Y,Smoking,Alcohol} | {LDL_3Y} | 0.083 | 0.909 | 1.185 | 0.092 | 0.767 | 0.546 |
| 189 | {BMI_2Y,WC_2Y,Alcohol} | {LDL_3Y} | 0.083 | 0.908 | 1.184 | 0.092 | 0.767 | 0.542 |
| 190 | {WC_2Y,Smoking,Alcohol} | {LDL_3Y} | 0.083 | 0.909 | 1.185 | 0.092 | 0.767 | 0.546 |
| 191 | {BP_2Y,WC_2Y,Alcohol} | {LDL_3Y} | 0.085 | 0.908 | 1.184 | 0.093 | 0.767 | 0.542 |
| 192 | {WC_2Y,Alcohol} | {LDL_3Y} | 0.085 | 0.908 | 1.184 | 0.093 | 0.767 | 0.542 |
| 193 | {BP_2Y,BMI_2Y,FBG_A1c_3Y,Smoking,Alcohol,Exercise} | {LDL_3Y} | 0.089 | 0.974 | 1.270 | 0.091 | 0.767 | 0.871 |
| 194 | {BMI_2Y,FBG_A1c_3Y,Smoking,Alcohol,Exercise} | {LDL_3Y} | 0.090 | 0.974 | 1.270 | 0.092 | 0.767 | 0.870 |
| 195 | {BP_2Y,BMI_2Y,FBG_A1c_3Y,Alcohol,Exercise} | {LDL_3Y} | 0.090 | 0.974 | 1.270 | 0.093 | 0.767 | 0.870 |
| 196 | {BP_2Y,BMI_2Y,Smoking,Alcohol,Exercise} | {LDL_3Y} | 0.091 | 0.905 | 1.180 | 0.101 | 0.767 | 0.526 |
| 197 | {BMI_2Y,FBG_A1c_3Y,Alcohol,Exercise} | {LDL_3Y} | 0.091 | 0.974 | 1.269 | 0.094 | 0.767 | 0.869 |
| 198 | {BMI_2Y,Smoking,Alcohol,Exercise} | {LDL_3Y} | 0.092 | 0.904 | 1.178 | 0.102 | 0.767 | 0.519 |
| 199 | {BP_2Y,BMI_2Y,Alcohol,Exercise} | {LDL_3Y} | 0.093 | 0.904 | 1.179 | 0.102 | 0.767 | 0.521 |
| 200 | {BMI_2Y,Alcohol,Exercise} | {LDL_3Y} | 0.093 | 0.903 | 1.177 | 0.103 | 0.767 | 0.514 |
| 201 | {BP_2Y,BMI_2Y,FBG_A1c_3Y,Smoking,Exercise} | {LDL_3Y} | 0.094 | 0.974 | 1.270 | 0.096 | 0.767 | 0.870 |
| 202 | {BMI_2Y,FBG_A1c_3Y,Smoking,Exercise} | {LDL_3Y} | 0.095 | 0.974 | 1.269 | 0.097 | 0.767 | 0.869 |
| 203 | {BP_2Y,BMI_2Y,Smoking,Exercise} | {LDL_3Y} | 0.096 | 0.905 | 1.179 | 0.106 | 0.767 | 0.524 |
| 204 | {BP_2Y,BMI_2Y,FBG_A1c_3Y,Exercise} | {LDL_3Y} | 0.096 | 0.973 | 1.269 | 0.099 | 0.767 | 0.866 |
| 205 | {BMI_2Y,Smoking,Exercise} | {LDL_3Y} | 0.097 | 0.903 | 1.178 | 0.107 | 0.767 | 0.517 |
| 206 | {BMI_2Y,FBG_A1c_3Y,Exercise} | {LDL_3Y} | 0.097 | 0.973 | 1.269 | 0.100 | 0.767 | 0.865 |
| 207 | {BP_2Y,BMI_2Y,WC_2Y,FBG_A1c_3Y,Smoking} | {LDL_3Y} | 0.098 | 0.976 | 1.272 | 0.100 | 0.767 | 0.879 |
| 208 | {BMI_2Y,WC_2Y,FBG_A1c_3Y,Smoking} | {LDL_3Y} | 0.098 | 0.976 | 1.272 | 0.101 | 0.767 | 0.879 |
| 209 | {BP_2Y,BMI_2Y,Exercise} | {LDL_3Y} | 0.099 | 0.903 | 1.177 | 0.110 | 0.767 | 0.513 |
| 210 | {BMI_2Y,Exercise} | {LDL_3Y} | 0.100 | 0.901 | 1.175 | 0.111 | 0.767 | 0.506 |
| 211 | {BP_2Y,BMI_2Y,WC_2Y,Smoking} | {LDL_3Y} | 0.100 | 0.915 | 1.193 | 0.109 | 0.767 | 0.575 |
| 212 | {BMI_2Y,WC_2Y,Smoking} | {LDL_3Y} | 0.100 | 0.915 | 1.193 | 0.110 | 0.767 | 0.574 |
| 213 | {BP_2Y,WC_2Y,FBG_A1c_3Y,Smoking} | {LDL_3Y} | 0.100 | 0.976 | 1.272 | 0.103 | 0.767 | 0.878 |
| 214 | {WC_2Y,FBG_A1c_3Y,Smoking} | {LDL_3Y} | 0.100 | 0.976 | 1.272 | 0.103 | 0.767 | 0.878 |
| 215 | {BP_2Y,WC_2Y,Smoking} | {LDL_3Y} | 0.102 | 0.915 | 1.192 | 0.112 | 0.767 | 0.573 |
| 216 | {WC_2Y,Smoking} | {LDL_3Y} | 0.103 | 0.915 | 1.192 | 0.112 | 0.767 | 0.573 |
| 217 | {BP_2Y,FBG_A1c_3Y,Smoking,Alcohol,Exercise} | {LDL_3Y} | 0.106 | 0.969 | 1.263 | 0.109 | 0.767 | 0.843 |
| 218 | {BP_2Y,BMI_2Y,WC_2Y,FBG_A1c_3Y} | {LDL_3Y} | 0.107 | 0.976 | 1.272 | 0.110 | 0.767 | 0.878 |
| 219 | {BMI_2Y,WC_2Y,FBG_A1c_3Y} | {LDL_3Y} | 0.107 | 0.976 | 1.272 | 0.110 | 0.767 | 0.878 |
| 220 | {BP_2Y,FBG_A1c_3Y,Alcohol,Exercise} | {LDL_3Y} | 0.107 | 0.968 | 1.262 | 0.111 | 0.767 | 0.840 |
| 221 | {BP_2Y,Smoking,Alcohol,Exercise} | {LDL_3Y} | 0.108 | 0.879 | 1.146 | 0.123 | 0.767 | 0.394 |
| 222 | {BP_2Y,BMI_2Y,WC_2Y} | {LDL_3Y} | 0.109 | 0.913 | 1.191 | 0.120 | 0.767 | 0.566 |
| 223 | {BMI_2Y,WC_2Y} | {LDL_3Y} | 0.110 | 0.913 | 1.190 | 0.120 | 0.767 | 0.565 |
| 224 | {BP_2Y,WC_2Y,FBG_A1c_3Y} | {LDL_3Y} | 0.110 | 0.975 | 1.272 | 0.113 | 0.767 | 0.877 |
| 225 | {WC_2Y,FBG_A1c_3Y} | {LDL_3Y} | 0.110 | 0.975 | 1.271 | 0.113 | 0.767 | 0.877 |
| 226 | {BP_2Y,Alcohol,Exercise} | {LDL_3Y} | 0.110 | 0.877 | 1.143 | 0.126 | 0.767 | 0.384 |
| 227 | {BP_2Y,FBG_A1c_3Y,Smoking,Exercise} | {LDL_3Y} | 0.112 | 0.967 | 1.261 | 0.116 | 0.767 | 0.837 |
| 228 | {BP_2Y,WC_2Y} | {LDL_3Y} | 0.112 | 0.913 | 1.190 | 0.123 | 0.767 | 0.565 |
| 229 | {WC_2Y} | {LDL_3Y} | 0.112 | 0.913 | 1.190 | 0.123 | 0.767 | 0.564 |
| 230 | {FBG_A1c_3Y,Smoking,Alcohol,Exercise} | {LDL_3Y} | 0.113 | 0.965 | 1.259 | 0.117 | 0.767 | 0.827 |
| 231 | {BP_2Y,Smoking,Exercise} | {LDL_3Y} | 0.115 | 0.876 | 1.142 | 0.131 | 0.767 | 0.379 |
| 232 | {BP_2Y,FBG_A1c_3Y,Exercise} | {LDL_3Y} | 0.115 | 0.966 | 1.260 | 0.119 | 0.767 | 0.831 |
| 233 | {FBG_A1c_3Y,Alcohol,Exercise} | {LDL_3Y} | 0.115 | 0.965 | 1.258 | 0.120 | 0.767 | 0.824 |
| 234 | {Smoking,Alcohol,Exercise} | {LDL_3Y} | 0.117 | 0.837 | 1.091 | 0.139 | 0.767 | 0.186 |
| 235 | {BP_2Y,Exercise} | {LDL_3Y} | 0.118 | 0.872 | 1.137 | 0.136 | 0.767 | 0.360 |
| 236 | {Alcohol,Exercise} | {LDL_3Y} | 0.119 | 0.835 | 1.089 | 0.142 | 0.767 | 0.175 |
| 237 | {FBG_A1c_3Y,Smoking,Exercise} | {LDL_3Y} | 0.120 | 0.964 | 1.257 | 0.125 | 0.767 | 0.821 |
| 238 | {Smoking,Exercise} | {LDL_3Y} | 0.124 | 0.834 | 1.088 | 0.148 | 0.767 | 0.172 |
| 239 | {FBG_A1c_3Y,Exercise} | {LDL_3Y} | 0.124 | 0.963 | 1.255 | 0.129 | 0.767 | 0.814 |
| 240 | {Exercise} | {LDL_3Y} | 0.127 | 0.831 | 1.083 | 0.153 | 0.767 | 0.153 |
| 241 | {BP_2Y,BMI_2Y,FBG_A1c_3Y,Smoking,Alcohol} | {LDL_3Y} | 0.345 | 0.969 | 1.263 | 0.355 | 0.767 | 0.846 |
| 242 | {BMI_2Y,FBG_A1c_3Y,Smoking,Alcohol} | {LDL_3Y} | 0.348 | 0.969 | 1.263 | 0.360 | 0.767 | 0.843 |
| 243 | {BP_2Y,BMI_2Y,FBG_A1c_3Y,Alcohol} | {LDL_3Y} | 0.351 | 0.968 | 1.262 | 0.362 | 0.767 | 0.842 |
| 244 | {BP_2Y,BMI_2Y,Smoking,Alcohol} | {LDL_3Y} | 0.353 | 0.897 | 1.170 | 0.394 | 0.767 | 0.487 |
| 245 | {BMI_2Y,FBG_A1c_3Y,Alcohol} | {LDL_3Y} | 0.355 | 0.968 | 1.262 | 0.366 | 0.767 | 0.840 |
| 246 | {BMI_2Y,Smoking,Alcohol} | {LDL_3Y} | 0.357 | 0.896 | 1.168 | 0.399 | 0.767 | 0.479 |
| 247 | {BP_2Y,BMI_2Y,Alcohol} | {LDL_3Y} | 0.360 | 0.896 | 1.168 | 0.401 | 0.767 | 0.480 |
| 248 | {BMI_2Y,Alcohol} | {LDL_3Y} | 0.364 | 0.894 | 1.166 | 0.407 | 0.767 | 0.471 |
| 249 | {BP_2Y,FBG_A1c_3Y,Smoking,Alcohol} | {LDL_3Y} | 0.424 | 0.961 | 1.253 | 0.441 | 0.767 | 0.807 |
| 250 | {BP_2Y,FBG_A1c_3Y,Alcohol} | {LDL_3Y} | 0.432 | 0.961 | 1.252 | 0.450 | 0.767 | 0.803 |
| 251 | {BP_2Y,Smoking,Alcohol} | {LDL_3Y} | 0.437 | 0.865 | 1.128 | 0.505 | 0.767 | 0.327 |
| 252 | {BP_2Y,Alcohol} | {LDL_3Y} | 0.445 | 0.864 | 1.126 | 0.516 | 0.767 | 0.318 |
| 253 | {BP_2Y,BMI_2Y,FBG_A1c_3Y,Smoking} | {LDL_3Y} | 0.456 | 0.966 | 1.260 | 0.471 | 0.767 | 0.832 |
| 254 | {FBG_A1c_3Y,Smoking,Alcohol} | {LDL_3Y} | 0.458 | 0.957 | 1.248 | 0.479 | 0.767 | 0.785 |
| 255 | {BMI_2Y,FBG_A1c_3Y,Smoking} | {LDL_3Y} | 0.461 | 0.966 | 1.259 | 0.477 | 0.767 | 0.828 |
| 256 | {BP_2Y,BMI_2Y,Smoking} | {LDL_3Y} | 0.467 | 0.892 | 1.162 | 0.524 | 0.767 | 0.458 |
| 257 | {FBG_A1c_3Y,Alcohol} | {LDL_3Y} | 0.467 | 0.956 | 1.246 | 0.489 | 0.767 | 0.780 |
| 258 | {Smoking,Alcohol} | {LDL_3Y} | 0.473 | 0.821 | 1.070 | 0.576 | 0.767 | 0.105 |
| 259 | {BMI_2Y,Smoking} | {LDL_3Y} | 0.473 | 0.889 | 1.159 | 0.532 | 0.767 | 0.446 |
| 260 | {Alcohol} | {LDL_3Y} | 0.482 | 0.818 | 1.067 | 0.589 | 0.767 | 0.092 |
| 261 | {BP_2Y,BMI_2Y,FBG_A1c_3Y} | {LDL_3Y} | 0.520 | 0.964 | 1.256 | 0.540 | 0.767 | 0.818 |
| 262 | {BMI_2Y,FBG_A1c_3Y} | {LDL_3Y} | 0.526 | 0.963 | 1.255 | 0.547 | 0.767 | 0.815 |
| 263 | {BP_2Y,BMI_2Y} | {LDL_3Y} | 0.535 | 0.884 | 1.153 | 0.605 | 0.767 | 0.421 |
| 264 | {BMI_2Y} | {LDL_3Y} | 0.541 | 0.882 | 1.149 | 0.614 | 0.767 | 0.408 |
| 265 | {BP_2Y,FBG_A1c_3Y,Smoking} | {LDL_3Y} | 0.572 | 0.957 | 1.248 | 0.598 | 0.767 | 0.787 |
| 266 | {BP_2Y,Smoking} | {LDL_3Y} | 0.589 | 0.856 | 1.116 | 0.689 | 0.767 | 0.280 |
| 267 | {FBG_A1c_3Y,Smoking} | {LDL_3Y} | 0.628 | 0.949 | 1.237 | 0.662 | 0.767 | 0.743 |
| 268 | {Smoking} | {LDL_3Y} | 0.648 | 0.805 | 1.049 | 0.804 | 0.767 | 0.032 |
| 269 | {BP_2Y,FBG_A1c_3Y} | {LDL_3Y} | 0.666 | 0.951 | 1.240 | 0.700 | 0.767 | 0.756 |
| 270 | {BP_2Y} | {LDL_3Y} | 0.687 | 0.838 | 1.093 | 0.820 | 0.767 | 0.282 |
| 271 | {FBG_A1c_3Y} | {LDL_3Y} | 0.741 | 0.941 | 1.226 | 0.788 | 0.767 | 0.810 |
| 272 | {BP_2Y,WC_2Y,LDL_3Y,Exercise} | {FBG_A1c_3Y} | 0.020 | 0.990 | 1.256 | 0.020 | 0.788 | 0.059 |
| 273 | {WC_2Y,LDL_3Y,Exercise} | {FBG_A1c_3Y} | 0.020 | 0.990 | 1.256 | 0.020 | 0.788 | 0.104 |
| 274 | {BP_2Y,BMI_2Y,WC_2Y,Exercise} | {FBG_A1c_3Y} | 0.020 | 0.914 | 1.160 | 0.022 | 0.788 | 0.034 |
| 275 | {BMI_2Y,WC_2Y,Exercise} | {FBG_A1c_3Y} | 0.020 | 0.914 | 1.160 | 0.022 | 0.788 | 0.040 |
| 276 | {Smoking,Diet,Exercise} | {FBG_A1c_3Y} | 0.020 | 0.854 | 1.084 | 0.024 | 0.788 | 0.037 |
| 277 | {BP_2Y,WC_2Y,Exercise} | {FBG_A1c_3Y} | 0.020 | 0.915 | 1.161 | 0.022 | 0.788 | 0.147 |
| 278 | {WC_2Y,Exercise} | {FBG_A1c_3Y} | 0.020 | 0.914 | 1.160 | 0.022 | 0.788 | 0.151 |
| 279 | {Diet,Exercise} | {FBG_A1c_3Y} | 0.021 | 0.851 | 1.081 | 0.024 | 0.788 | 0.130 |
| 280 | {BP_2Y,BMI_2Y,LDL_3Y,Smoking,Alcohol,Diet} | {FBG_A1c_3Y} | 0.023 | 0.989 | 1.256 | 0.023 | 0.788 | 0.924 |
| 281 | {BMI_2Y,LDL_3Y,Smoking,Alcohol,Diet} | {FBG_A1c_3Y} | 0.023 | 0.989 | 1.255 | 0.023 | 0.788 | 0.924 |
| 282 | {BP_2Y,BMI_2Y,LDL_3Y,Alcohol,Diet} | {FBG_A1c_3Y} | 0.023 | 0.989 | 1.255 | 0.024 | 0.788 | 0.927 |
| 283 | {BMI_2Y,LDL_3Y,Alcohol,Diet} | {FBG_A1c_3Y} | 0.023 | 0.989 | 1.255 | 0.024 | 0.788 | 0.927 |
| 284 | {BP_2Y,BMI_2Y,Smoking,Alcohol,Diet} | {FBG_A1c_3Y} | 0.024 | 0.918 | 1.166 | 0.026 | 0.788 | 0.592 |
| 285 | {BP_2Y,BMI_2Y,LDL_3Y,Smoking,Diet} | {FBG_A1c_3Y} | 0.024 | 0.990 | 1.256 | 0.024 | 0.788 | 0.940 |
| 286 | {BMI_2Y,Smoking,Alcohol,Diet} | {FBG_A1c_3Y} | 0.024 | 0.917 | 1.164 | 0.026 | 0.788 | 0.586 |
| 287 | {BMI_2Y,LDL_3Y,Smoking,Diet} | {FBG_A1c_3Y} | 0.024 | 0.989 | 1.256 | 0.024 | 0.788 | 0.940 |
| 288 | {BP_2Y,BMI_2Y,Alcohol,Diet} | {FBG_A1c_3Y} | 0.024 | 0.917 | 1.164 | 0.026 | 0.788 | 0.584 |
| 289 | {BMI_2Y,Alcohol,Diet} | {FBG_A1c_3Y} | 0.024 | 0.915 | 1.162 | 0.027 | 0.788 | 0.577 |
| 290 | {BP_2Y,BMI_2Y,LDL_3Y,Diet} | {FBG_A1c_3Y} | 0.024 | 0.989 | 1.255 | 0.025 | 0.788 | 0.940 |
| 291 | {BMI_2Y,LDL_3Y,Diet} | {FBG_A1c_3Y} | 0.025 | 0.988 | 1.254 | 0.025 | 0.788 | 0.940 |
| 292 | {BP_2Y,BMI_2Y,Smoking,Diet} | {FBG_A1c_3Y} | 0.025 | 0.919 | 1.166 | 0.027 | 0.788 | 0.595 |
| 293 | {BMI_2Y,Smoking,Diet} | {FBG_A1c_3Y} | 0.025 | 0.918 | 1.165 | 0.027 | 0.788 | 0.588 |
| 294 | {BP_2Y,BMI_2Y,Diet} | {FBG_A1c_3Y} | 0.025 | 0.917 | 1.164 | 0.028 | 0.788 | 0.584 |
| 295 | {BMI_2Y,Diet} | {FBG_A1c_3Y} | 0.025 | 0.915 | 1.162 | 0.028 | 0.788 | 0.576 |
| 296 | {BP_2Y,LDL_3Y,Smoking,Alcohol,Diet} | {FBG_A1c_3Y} | 0.026 | 0.986 | 1.252 | 0.026 | 0.788 | 0.932 |
| 297 | {BP_2Y,LDL_3Y,Alcohol,Diet} | {FBG_A1c_3Y} | 0.026 | 0.986 | 1.251 | 0.027 | 0.788 | 0.928 |
| 298 | {BP_2Y,Smoking,Alcohol,Diet} | {FBG_A1c_3Y} | 0.027 | 0.893 | 1.134 | 0.030 | 0.788 | 0.467 |
| 299 | {BP_2Y,LDL_3Y,Smoking,Diet} | {FBG_A1c_3Y} | 0.027 | 0.986 | 1.252 | 0.027 | 0.788 | 0.932 |
| 300 | {LDL_3Y,Smoking,Alcohol,Diet} | {FBG_A1c_3Y} | 0.027 | 0.985 | 1.251 | 0.027 | 0.788 | 0.927 |
| 301 | {BP_2Y,Alcohol,Diet} | {FBG_A1c_3Y} | 0.027 | 0.891 | 1.130 | 0.030 | 0.788 | 0.453 |
| 302 | {LDL_3Y,Alcohol,Diet} | {FBG_A1c_3Y} | 0.027 | 0.985 | 1.250 | 0.028 | 0.788 | 0.924 |
| 303 | {BP_2Y,LDL_3Y,Diet} | {FBG_A1c_3Y} | 0.028 | 0.985 | 1.251 | 0.028 | 0.788 | 0.926 |
| 304 | {BP_2Y,Smoking,Diet} | {FBG_A1c_3Y} | 0.028 | 0.893 | 1.134 | 0.031 | 0.788 | 0.466 |
| 305 | {LDL_3Y,Smoking,Diet} | {FBG_A1c_3Y} | 0.028 | 0.986 | 1.251 | 0.029 | 0.788 | 0.928 |
| 306 | {Smoking,Alcohol,Diet} | {FBG_A1c_3Y} | 0.028 | 0.840 | 1.066 | 0.034 | 0.788 | 0.199 |
| 307 | {BP_2Y,Diet} | {FBG_A1c_3Y} | 0.029 | 0.890 | 1.130 | 0.032 | 0.788 | 0.450 |
| 308 | {Alcohol,Diet} | {FBG_A1c_3Y} | 0.029 | 0.837 | 1.062 | 0.034 | 0.788 | 0.184 |
| 309 | {LDL_3Y,Diet} | {FBG_A1c_3Y} | 0.029 | 0.984 | 1.250 | 0.029 | 0.788 | 0.922 |
| 310 | {Smoking,Diet} | {FBG_A1c_3Y} | 0.029 | 0.840 | 1.066 | 0.035 | 0.788 | 0.198 |
| 311 | {Diet} | {FBG_A1c_3Y} | 0.030 | 0.837 | 1.062 | 0.036 | 0.788 | 0.184 |
| 312 | {BP_2Y,BMI_2Y,WC_2Y,LDL_3Y,Smoking,Alcohol} | {FBG_A1c_3Y} | 0.080 | 0.977 | 1.240 | 0.082 | 0.788 | 0.885 |
| 313 | {BMI_2Y,WC_2Y,LDL_3Y,Smoking,Alcohol} | {FBG_A1c_3Y} | 0.080 | 0.977 | 1.240 | 0.082 | 0.788 | 0.883 |
| 314 | {BP_2Y,BMI_2Y,WC_2Y,LDL_3Y,Alcohol} | {FBG_A1c_3Y} | 0.082 | 0.977 | 1.240 | 0.083 | 0.788 | 0.886 |
| 315 | {BP_2Y,WC_2Y,LDL_3Y,Smoking,Alcohol} | {FBG_A1c_3Y} | 0.082 | 0.977 | 1.240 | 0.083 | 0.788 | 0.886 |
| 316 | {BMI_2Y,WC_2Y,LDL_3Y,Alcohol} | {FBG_A1c_3Y} | 0.082 | 0.977 | 1.240 | 0.083 | 0.788 | 0.884 |
| 317 | {WC_2Y,LDL_3Y,Smoking,Alcohol} | {FBG_A1c_3Y} | 0.082 | 0.977 | 1.240 | 0.083 | 0.788 | 0.885 |
| 318 | {BP_2Y,BMI_2Y,WC_2Y,Smoking,Alcohol} | {FBG_A1c_3Y} | 0.082 | 0.910 | 1.156 | 0.091 | 0.788 | 0.552 |
| 319 | {BMI_2Y,WC_2Y,Smoking,Alcohol} | {FBG_A1c_3Y} | 0.082 | 0.910 | 1.155 | 0.091 | 0.788 | 0.551 |
| 320 | {BP_2Y,WC_2Y,LDL_3Y,Alcohol} | {FBG_A1c_3Y} | 0.083 | 0.977 | 1.241 | 0.085 | 0.788 | 0.887 |
| 321 | {WC_2Y,LDL_3Y,Alcohol} | {FBG_A1c_3Y} | 0.083 | 0.977 | 1.240 | 0.085 | 0.788 | 0.886 |
| 322 | {BP_2Y,BMI_2Y,WC_2Y,Alcohol} | {FBG_A1c_3Y} | 0.084 | 0.910 | 1.155 | 0.092 | 0.788 | 0.550 |
| 323 | {BP_2Y,WC_2Y,Smoking,Alcohol} | {FBG_A1c_3Y} | 0.084 | 0.911 | 1.156 | 0.092 | 0.788 | 0.554 |
| 324 | {BMI_2Y,WC_2Y,Alcohol} | {FBG_A1c_3Y} | 0.084 | 0.910 | 1.155 | 0.092 | 0.788 | 0.549 |
| 325 | {WC_2Y,Smoking,Alcohol} | {FBG_A1c_3Y} | 0.084 | 0.911 | 1.156 | 0.092 | 0.788 | 0.553 |
| 326 | {BP_2Y,WC_2Y,Alcohol} | {FBG_A1c_3Y} | 0.085 | 0.910 | 1.155 | 0.093 | 0.788 | 0.551 |
| 327 | {WC_2Y,Alcohol} | {FBG_A1c_3Y} | 0.085 | 0.910 | 1.155 | 0.093 | 0.788 | 0.550 |
| 328 | {BP_2Y,BMI_2Y,LDL_3Y,Smoking,Alcohol,Exercise} | {FBG_A1c_3Y} | 0.089 | 0.975 | 1.238 | 0.091 | 0.788 | 0.877 |
| 329 | {BMI_2Y,LDL_3Y,Smoking,Alcohol,Exercise} | {FBG_A1c_3Y} | 0.090 | 0.975 | 1.238 | 0.092 | 0.788 | 0.876 |
| 330 | {BP_2Y,BMI_2Y,LDL_3Y,Alcohol,Exercise} | {FBG_A1c_3Y} | 0.090 | 0.975 | 1.238 | 0.093 | 0.788 | 0.875 |
| 331 | {BMI_2Y,LDL_3Y,Alcohol,Exercise} | {FBG_A1c_3Y} | 0.091 | 0.975 | 1.238 | 0.093 | 0.788 | 0.875 |
| 332 | {BP_2Y,BMI_2Y,Smoking,Alcohol,Exercise} | {FBG_A1c_3Y} | 0.091 | 0.906 | 1.150 | 0.101 | 0.788 | 0.531 |
| 333 | {BMI_2Y,Smoking,Alcohol,Exercise} | {FBG_A1c_3Y} | 0.092 | 0.905 | 1.149 | 0.102 | 0.788 | 0.525 |
| 334 | {BP_2Y,BMI_2Y,Alcohol,Exercise} | {FBG_A1c_3Y} | 0.093 | 0.905 | 1.149 | 0.102 | 0.788 | 0.525 |
| 335 | {BMI_2Y,Alcohol,Exercise} | {FBG_A1c_3Y} | 0.094 | 0.904 | 1.147 | 0.103 | 0.788 | 0.520 |
| 336 | {BP_2Y,BMI_2Y,LDL_3Y,Smoking,Exercise} | {FBG_A1c_3Y} | 0.094 | 0.976 | 1.239 | 0.096 | 0.788 | 0.881 |
| 337 | {BMI_2Y,LDL_3Y,Smoking,Exercise} | {FBG_A1c_3Y} | 0.095 | 0.976 | 1.239 | 0.097 | 0.788 | 0.881 |
| 338 | {BP_2Y,BMI_2Y,Smoking,Exercise} | {FBG_A1c_3Y} | 0.096 | 0.907 | 1.151 | 0.106 | 0.788 | 0.534 |
| 339 | {BP_2Y,BMI_2Y,LDL_3Y,Exercise} | {FBG_A1c_3Y} | 0.096 | 0.976 | 1.238 | 0.099 | 0.788 | 0.878 |
| 340 | {BMI_2Y,Smoking,Exercise} | {FBG_A1c_3Y} | 0.097 | 0.906 | 1.149 | 0.107 | 0.788 | 0.528 |
| 341 | {BMI_2Y,LDL_3Y,Exercise} | {FBG_A1c_3Y} | 0.097 | 0.976 | 1.238 | 0.100 | 0.788 | 0.878 |
| 342 | {BP_2Y,BMI_2Y,WC_2Y,LDL_3Y,Smoking} | {FBG_A1c_3Y} | 0.098 | 0.979 | 1.243 | 0.100 | 0.788 | 0.895 |
| 343 | {BMI_2Y,WC_2Y,LDL_3Y,Smoking} | {FBG_A1c_3Y} | 0.098 | 0.979 | 1.242 | 0.100 | 0.788 | 0.894 |
| 344 | {BP_2Y,BMI_2Y,Exercise} | {FBG_A1c_3Y} | 0.099 | 0.905 | 1.149 | 0.110 | 0.788 | 0.524 |
| 345 | {BMI_2Y,Exercise} | {FBG_A1c_3Y} | 0.100 | 0.904 | 1.147 | 0.111 | 0.788 | 0.518 |
| 346 | {BP_2Y,WC_2Y,LDL_3Y,Smoking} | {FBG_A1c_3Y} | 0.100 | 0.979 | 1.243 | 0.102 | 0.788 | 0.897 |
| 347 | {WC_2Y,LDL_3Y,Smoking} | {FBG_A1c_3Y} | 0.100 | 0.979 | 1.243 | 0.103 | 0.788 | 0.896 |
| 348 | {BP_2Y,BMI_2Y,WC_2Y,Smoking} | {FBG_A1c_3Y} | 0.100 | 0.918 | 1.165 | 0.109 | 0.788 | 0.589 |
| 349 | {BMI_2Y,WC_2Y,Smoking} | {FBG_A1c_3Y} | 0.101 | 0.918 | 1.165 | 0.110 | 0.788 | 0.588 |
| 350 | {BP_2Y,WC_2Y,Smoking} | {FBG_A1c_3Y} | 0.103 | 0.918 | 1.165 | 0.112 | 0.788 | 0.591 |
| 351 | {WC_2Y,Smoking} | {FBG_A1c_3Y} | 0.103 | 0.918 | 1.165 | 0.112 | 0.788 | 0.590 |
| 352 | {BP_2Y,LDL_3Y,Smoking,Alcohol,Exercise} | {FBG_A1c_3Y} | 0.106 | 0.973 | 1.235 | 0.108 | 0.788 | 0.867 |
| 353 | {BP_2Y,BMI_2Y,WC_2Y,LDL_3Y} | {FBG_A1c_3Y} | 0.107 | 0.979 | 1.243 | 0.109 | 0.788 | 0.897 |
| 354 | {BMI_2Y,WC_2Y,LDL_3Y} | {FBG_A1c_3Y} | 0.107 | 0.979 | 1.243 | 0.110 | 0.788 | 0.897 |
| 355 | {BP_2Y,LDL_3Y,Alcohol,Exercise} | {FBG_A1c_3Y} | 0.107 | 0.973 | 1.235 | 0.110 | 0.788 | 0.865 |
| 356 | {BP_2Y,Smoking,Alcohol,Exercise} | {FBG_A1c_3Y} | 0.109 | 0.883 | 1.121 | 0.123 | 0.788 | 0.416 |
| 357 | {BP_2Y,BMI_2Y,WC_2Y} | {FBG_A1c_3Y} | 0.110 | 0.917 | 1.164 | 0.120 | 0.788 | 0.584 |
| 358 | {BMI_2Y,WC_2Y} | {FBG_A1c_3Y} | 0.110 | 0.917 | 1.163 | 0.120 | 0.788 | 0.583 |
| 359 | {BP_2Y,WC_2Y,LDL_3Y} | {FBG_A1c_3Y} | 0.110 | 0.980 | 1.244 | 0.112 | 0.788 | 0.900 |
| 360 | {WC_2Y,LDL_3Y} | {FBG_A1c_3Y} | 0.110 | 0.980 | 1.244 | 0.112 | 0.788 | 0.899 |
| 361 | {BP_2Y,Alcohol,Exercise} | {FBG_A1c_3Y} | 0.111 | 0.881 | 1.119 | 0.126 | 0.788 | 0.406 |
| 362 | {BP_2Y,LDL_3Y,Smoking,Exercise} | {FBG_A1c_3Y} | 0.112 | 0.974 | 1.237 | 0.115 | 0.788 | 0.871 |
| 363 | {BP_2Y,WC_2Y} | {FBG_A1c_3Y} | 0.113 | 0.917 | 1.164 | 0.123 | 0.788 | 0.587 |
| 364 | {WC_2Y} | {FBG_A1c_3Y} | 0.113 | 0.917 | 1.164 | 0.123 | 0.788 | 0.585 |
| 365 | {LDL_3Y,Smoking,Alcohol,Exercise} | {FBG_A1c_3Y} | 0.113 | 0.972 | 1.234 | 0.117 | 0.788 | 0.861 |
| 366 | {BP_2Y,LDL_3Y,Exercise} | {FBG_A1c_3Y} | 0.115 | 0.974 | 1.236 | 0.118 | 0.788 | 0.868 |
| 367 | {LDL_3Y,Alcohol,Exercise} | {FBG_A1c_3Y} | 0.115 | 0.972 | 1.234 | 0.119 | 0.788 | 0.859 |
| 368 | {BP_2Y,Smoking,Exercise} | {FBG_A1c_3Y} | 0.116 | 0.882 | 1.119 | 0.131 | 0.788 | 0.410 |
| 369 | {Smoking,Alcohol,Exercise} | {FBG_A1c_3Y} | 0.117 | 0.843 | 1.070 | 0.139 | 0.788 | 0.215 |
| 370 | {BP_2Y,Exercise} | {FBG_A1c_3Y} | 0.119 | 0.879 | 1.115 | 0.136 | 0.788 | 0.394 |
| 371 | {Alcohol,Exercise} | {FBG_A1c_3Y} | 0.120 | 0.841 | 1.068 | 0.142 | 0.788 | 0.206 |
| 372 | {LDL_3Y,Smoking,Exercise} | {FBG_A1c_3Y} | 0.120 | 0.973 | 1.235 | 0.124 | 0.788 | 0.865 |
| 373 | {LDL_3Y,Exercise} | {FBG_A1c_3Y} | 0.124 | 0.972 | 1.234 | 0.127 | 0.788 | 0.861 |
| 374 | {Smoking,Exercise} | {FBG_A1c_3Y} | 0.125 | 0.842 | 1.069 | 0.148 | 0.788 | 0.210 |
| 375 | {Exercise} | {FBG_A1c_3Y} | 0.129 | 0.839 | 1.064 | 0.153 | 0.788 | 0.193 |
| 376 | {BP_2Y,BMI_2Y,LDL_3Y,Smoking,Alcohol} | {FBG_A1c_3Y} | 0.345 | 0.975 | 1.238 | 0.353 | 0.788 | 0.876 |
| 377 | {BMI_2Y,LDL_3Y,Smoking,Alcohol} | {FBG_A1c_3Y} | 0.348 | 0.975 | 1.238 | 0.357 | 0.788 | 0.875 |
| 378 | {BP_2Y,BMI_2Y,LDL_3Y,Alcohol} | {FBG_A1c_3Y} | 0.351 | 0.975 | 1.237 | 0.360 | 0.788 | 0.874 |
| 379 | {BMI_2Y,LDL_3Y,Alcohol} | {FBG_A1c_3Y} | 0.355 | 0.975 | 1.237 | 0.364 | 0.788 | 0.874 |
| 380 | {BP_2Y,BMI_2Y,Smoking,Alcohol} | {FBG_A1c_3Y} | 0.355 | 0.903 | 1.146 | 0.394 | 0.788 | 0.515 |
| 381 | {BMI_2Y,Smoking,Alcohol} | {FBG_A1c_3Y} | 0.360 | 0.902 | 1.145 | 0.399 | 0.788 | 0.509 |
| 382 | {BP_2Y,BMI_2Y,Alcohol} | {FBG_A1c_3Y} | 0.362 | 0.902 | 1.145 | 0.401 | 0.788 | 0.510 |
| 383 | {BMI_2Y,Alcohol} | {FBG_A1c_3Y} | 0.366 | 0.901 | 1.143 | 0.407 | 0.788 | 0.503 |
| 384 | {BP_2Y,LDL_3Y,Smoking,Alcohol} | {FBG_A1c_3Y} | 0.424 | 0.971 | 1.233 | 0.437 | 0.788 | 0.856 |
| 385 | {BP_2Y,LDL_3Y,Alcohol} | {FBG_A1c_3Y} | 0.432 | 0.971 | 1.232 | 0.445 | 0.788 | 0.854 |
| 386 | {BP_2Y,Smoking,Alcohol} | {FBG_A1c_3Y} | 0.441 | 0.874 | 1.110 | 0.505 | 0.788 | 0.371 |
| 387 | {BP_2Y,Alcohol} | {FBG_A1c_3Y} | 0.450 | 0.873 | 1.108 | 0.516 | 0.788 | 0.364 |
| 388 | {BP_2Y,BMI_2Y,LDL_3Y,Smoking} | {FBG_A1c_3Y} | 0.456 | 0.975 | 1.238 | 0.467 | 0.788 | 0.875 |
| 389 | {LDL_3Y,Smoking,Alcohol} | {FBG_A1c_3Y} | 0.458 | 0.970 | 1.231 | 0.473 | 0.788 | 0.848 |
| 390 | {BMI_2Y,LDL_3Y,Smoking} | {FBG_A1c_3Y} | 0.461 | 0.975 | 1.237 | 0.473 | 0.788 | 0.874 |
| 391 | {LDL_3Y,Alcohol} | {FBG_A1c_3Y} | 0.467 | 0.969 | 1.230 | 0.482 | 0.788 | 0.847 |
| 392 | {BP_2Y,BMI_2Y,Smoking} | {FBG_A1c_3Y} | 0.471 | 0.900 | 1.142 | 0.524 | 0.788 | 0.498 |
| 393 | {BMI_2Y,Smoking} | {FBG_A1c_3Y} | 0.477 | 0.898 | 1.139 | 0.532 | 0.788 | 0.488 |
| 394 | {Smoking,Alcohol} | {FBG_A1c_3Y} | 0.479 | 0.832 | 1.056 | 0.576 | 0.788 | 0.160 |
| 395 | {Alcohol} | {FBG_A1c_3Y} | 0.489 | 0.830 | 1.053 | 0.589 | 0.788 | 0.149 |
| 396 | {BP_2Y,BMI_2Y,LDL_3Y} | {FBG_A1c_3Y} | 0.520 | 0.973 | 1.235 | 0.535 | 0.788 | 0.864 |
| 397 | {BMI_2Y,LDL_3Y} | {FBG_A1c_3Y} | 0.526 | 0.973 | 1.235 | 0.541 | 0.788 | 0.863 |
| 398 | {BP_2Y,BMI_2Y} | {FBG_A1c_3Y} | 0.540 | 0.893 | 1.133 | 0.605 | 0.788 | 0.463 |
| 399 | {BMI_2Y} | {FBG_A1c_3Y} | 0.547 | 0.891 | 1.130 | 0.614 | 0.788 | 0.453 |
| 400 | {BP_2Y,LDL_3Y,Smoking} | {FBG_A1c_3Y} | 0.572 | 0.971 | 1.232 | 0.589 | 0.788 | 0.855 |
| 401 | {BP_2Y,Smoking} | {FBG_A1c_3Y} | 0.598 | 0.868 | 1.102 | 0.689 | 0.788 | 0.341 |
| 402 | {LDL_3Y,Smoking} | {FBG_A1c_3Y} | 0.628 | 0.969 | 1.231 | 0.648 | 0.788 | 0.847 |
| 403 | {Smoking} | {FBG_A1c_3Y} | 0.662 | 0.823 | 1.044 | 0.804 | 0.788 | 0.126 |
| 404 | {BP_2Y,LDL_3Y} | {FBG_A1c_3Y} | 0.666 | 0.968 | 1.229 | 0.687 | 0.788 | 0.842 |
| 405 | {BP_2Y} | {FBG_A1c_3Y} | 0.700 | 0.853 | 1.083 | 0.820 | 0.788 | 0.333 |
| 406 | {LDL_3Y} | {FBG_A1c_3Y} | 0.741 | 0.966 | 1.226 | 0.767 | 0.788 | 0.831 |
| 407 | {BP_2Y,WC_2Y,FBG_A1c_3Y,Exercise} | {BMI_2Y} | 0.020 | 0.987 | 1.608 | 0.020 | 0.614 | 0.205 |
| 408 | {WC_2Y,FBG_A1c_3Y,Exercise} | {BMI_2Y} | 0.020 | 0.987 | 1.608 | 0.020 | 0.614 | 0.231 |
| 409 | {BP_2Y,WC_2Y,Smoking,Exercise} | {BMI_2Y} | 0.021 | 0.988 | 1.610 | 0.022 | 0.614 | 0.837 |
| 410 | {WC_2Y,Smoking,Exercise} | {BMI_2Y} | 0.021 | 0.988 | 1.610 | 0.022 | 0.614 | 0.839 |
| 411 | {BP_2Y,WC_2Y,Exercise} | {BMI_2Y} | 0.022 | 0.988 | 1.609 | 0.022 | 0.614 | 0.878 |
| 412 | {WC_2Y,Exercise} | {BMI_2Y} | 0.022 | 0.988 | 1.609 | 0.022 | 0.614 | 0.879 |
| 413 | {BP_2Y,LDL_3Y,FBG_A1c_3Y,Smoking,Alcohol,Diet} | {BMI_2Y} | 0.023 | 0.892 | 1.453 | 0.026 | 0.614 | 0.462 |
| 414 | {LDL_3Y,FBG_A1c_3Y,Smoking,Alcohol,Diet} | {BMI_2Y} | 0.023 | 0.855 | 1.393 | 0.027 | 0.614 | 0.276 |
| 415 | {BP_2Y,LDL_3Y,Smoking,Alcohol,Diet} | {BMI_2Y} | 0.023 | 0.890 | 1.449 | 0.026 | 0.614 | 0.448 |
| 416 | {BP_2Y,LDL_3Y,FBG_A1c_3Y,Alcohol,Diet} | {BMI_2Y} | 0.023 | 0.892 | 1.452 | 0.026 | 0.614 | 0.459 |
| 417 | {LDL_3Y,Smoking,Alcohol,Diet} | {BMI_2Y} | 0.023 | 0.852 | 1.388 | 0.027 | 0.614 | 0.261 |
| 418 | {LDL_3Y,FBG_A1c_3Y,Alcohol,Diet} | {BMI_2Y} | 0.023 | 0.855 | 1.392 | 0.027 | 0.614 | 0.274 |
| 419 | {BP_2Y,LDL_3Y,Alcohol,Diet} | {BMI_2Y} | 0.024 | 0.889 | 1.448 | 0.027 | 0.614 | 0.444 |
| 420 | {LDL_3Y,Alcohol,Diet} | {BMI_2Y} | 0.024 | 0.852 | 1.387 | 0.028 | 0.614 | 0.258 |
| 421 | {BP_2Y,FBG_A1c_3Y,Smoking,Alcohol,Diet} | {BMI_2Y} | 0.024 | 0.890 | 1.450 | 0.027 | 0.614 | 0.450 |
| 422 | {BP_2Y,LDL_3Y,FBG_A1c_3Y,Smoking,Diet} | {BMI_2Y} | 0.024 | 0.891 | 1.451 | 0.027 | 0.614 | 0.455 |
| 423 | {FBG_A1c_3Y,Smoking,Alcohol,Diet} | {BMI_2Y} | 0.024 | 0.849 | 1.383 | 0.028 | 0.614 | 0.244 |
| 424 | {LDL_3Y,FBG_A1c_3Y,Smoking,Diet} | {BMI_2Y} | 0.024 | 0.854 | 1.391 | 0.028 | 0.614 | 0.270 |
| 425 | {BP_2Y,FBG_A1c_3Y,Alcohol,Diet} | {BMI_2Y} | 0.024 | 0.890 | 1.449 | 0.027 | 0.614 | 0.448 |
| 426 | {BP_2Y,LDL_3Y,Smoking,Diet} | {BMI_2Y} | 0.024 | 0.888 | 1.446 | 0.027 | 0.614 | 0.440 |
| 427 | {FBG_A1c_3Y,Alcohol,Diet} | {BMI_2Y} | 0.024 | 0.848 | 1.382 | 0.029 | 0.614 | 0.242 |
| 428 | {LDL_3Y,Smoking,Diet} | {BMI_2Y} | 0.024 | 0.851 | 1.386 | 0.029 | 0.614 | 0.254 |
| 429 | {BP_2Y,LDL_3Y,FBG_A1c_3Y,Diet} | {BMI_2Y} | 0.024 | 0.887 | 1.445 | 0.028 | 0.614 | 0.436 |
| 430 | {LDL_3Y,FBG_A1c_3Y,Diet} | {BMI_2Y} | 0.025 | 0.851 | 1.385 | 0.029 | 0.614 | 0.253 |
| 431 | {BP_2Y,LDL_3Y,Diet} | {BMI_2Y} | 0.025 | 0.884 | 1.440 | 0.028 | 0.614 | 0.421 |
| 432 | {BP_2Y,FBG_A1c_3Y,Smoking,Diet} | {BMI_2Y} | 0.025 | 0.888 | 1.447 | 0.028 | 0.614 | 0.442 |
| 433 | {LDL_3Y,Diet} | {BMI_2Y} | 0.025 | 0.847 | 1.380 | 0.029 | 0.614 | 0.237 |
| 434 | {FBG_A1c_3Y,Smoking,Diet} | {BMI_2Y} | 0.025 | 0.848 | 1.381 | 0.029 | 0.614 | 0.238 |
| 435 | {BP_2Y,FBG_A1c_3Y,Diet} | {BMI_2Y} | 0.025 | 0.885 | 1.441 | 0.029 | 0.614 | 0.424 |
| 436 | {FBG_A1c_3Y,Diet} | {BMI_2Y} | 0.025 | 0.844 | 1.374 | 0.030 | 0.614 | 0.219 |
| 437 | {BP_2Y,Smoking,Alcohol,Diet} | {BMI_2Y} | 0.026 | 0.866 | 1.410 | 0.030 | 0.614 | 0.329 |
| 438 | {BP_2Y,Alcohol,Diet} | {BMI_2Y} | 0.026 | 0.864 | 1.408 | 0.030 | 0.614 | 0.321 |
| 439 | {BP_2Y,Smoking,Diet} | {BMI_2Y} | 0.027 | 0.864 | 1.406 | 0.031 | 0.614 | 0.318 |
| 440 | {BP_2Y,Diet} | {BMI_2Y} | 0.028 | 0.859 | 1.399 | 0.032 | 0.614 | 0.295 |
| 441 | {BP_2Y,WC_2Y,LDL_3Y,FBG_A1c_3Y,Smoking,Alcohol} | {BMI_2Y} | 0.080 | 0.987 | 1.607 | 0.082 | 0.614 | 0.933 |
| 442 | {WC_2Y,LDL_3Y,FBG_A1c_3Y,Smoking,Alcohol} | {BMI_2Y} | 0.080 | 0.987 | 1.607 | 0.082 | 0.614 | 0.933 |
| 443 | {BP_2Y,WC_2Y,LDL_3Y,FBG_A1c_3Y,Alcohol} | {BMI_2Y} | 0.082 | 0.987 | 1.607 | 0.083 | 0.614 | 0.933 |
| 444 | {WC_2Y,LDL_3Y,FBG_A1c_3Y,Alcohol} | {BMI_2Y} | 0.082 | 0.987 | 1.607 | 0.083 | 0.614 | 0.933 |
| 445 | {BP_2Y,WC_2Y,LDL_3Y,Smoking,Alcohol} | {BMI_2Y} | 0.082 | 0.987 | 1.607 | 0.083 | 0.614 | 0.934 |
| 446 | {WC_2Y,LDL_3Y,Smoking,Alcohol} | {BMI_2Y} | 0.082 | 0.987 | 1.607 | 0.083 | 0.614 | 0.934 |
| 447 | {BP_2Y,WC_2Y,FBG_A1c_3Y,Smoking,Alcohol} | {BMI_2Y} | 0.082 | 0.986 | 1.607 | 0.084 | 0.614 | 0.932 |
| 448 | {WC_2Y,FBG_A1c_3Y,Smoking,Alcohol} | {BMI_2Y} | 0.082 | 0.987 | 1.607 | 0.084 | 0.614 | 0.933 |
| 449 | {BP_2Y,WC_2Y,LDL_3Y,Alcohol} | {BMI_2Y} | 0.083 | 0.987 | 1.607 | 0.085 | 0.614 | 0.935 |
| 450 | {WC_2Y,LDL_3Y,Alcohol} | {BMI_2Y} | 0.083 | 0.987 | 1.607 | 0.085 | 0.614 | 0.935 |
| 451 | {BP_2Y,WC_2Y,FBG_A1c_3Y,Alcohol} | {BMI_2Y} | 0.084 | 0.987 | 1.607 | 0.085 | 0.614 | 0.933 |
| 452 | {WC_2Y,FBG_A1c_3Y,Alcohol} | {BMI_2Y} | 0.084 | 0.987 | 1.607 | 0.085 | 0.614 | 0.933 |
| 453 | {BP_2Y,LDL_3Y,FBG_A1c_3Y,Smoking,Alcohol,Exercise} | {BMI_2Y} | 0.089 | 0.842 | 1.371 | 0.106 | 0.614 | 0.208 |
| 454 | {BP_2Y,LDL_3Y,FBG_A1c_3Y,Alcohol,Exercise} | {BMI_2Y} | 0.090 | 0.841 | 1.370 | 0.107 | 0.614 | 0.206 |
| 455 | {BP_2Y,WC_2Y,Smoking,Alcohol} | {BMI_2Y} | 0.091 | 0.987 | 1.607 | 0.092 | 0.614 | 0.934 |
| 456 | {WC_2Y,Smoking,Alcohol} | {BMI_2Y} | 0.091 | 0.987 | 1.607 | 0.092 | 0.614 | 0.934 |
| 457 | {BP_2Y,LDL_3Y,Smoking,Alcohol,Exercise} | {BMI_2Y} | 0.091 | 0.840 | 1.368 | 0.108 | 0.614 | 0.199 |
| 458 | {BP_2Y,FBG_A1c_3Y,Smoking,Alcohol,Exercise} | {BMI_2Y} | 0.091 | 0.837 | 1.362 | 0.109 | 0.614 | 0.183 |
| 459 | {BP_2Y,WC_2Y,Alcohol} | {BMI_2Y} | 0.092 | 0.987 | 1.607 | 0.093 | 0.614 | 0.934 |
| 460 | {WC_2Y,Alcohol} | {BMI_2Y} | 0.092 | 0.987 | 1.607 | 0.093 | 0.614 | 0.934 |
| 461 | {BP_2Y,LDL_3Y,Alcohol,Exercise} | {BMI_2Y} | 0.093 | 0.840 | 1.367 | 0.110 | 0.614 | 0.198 |
| 462 | {BP_2Y,FBG_A1c_3Y,Alcohol,Exercise} | {BMI_2Y} | 0.093 | 0.836 | 1.362 | 0.111 | 0.614 | 0.181 |
| 463 | {BP_2Y,LDL_3Y,FBG_A1c_3Y,Smoking,Exercise} | {BMI_2Y} | 0.094 | 0.837 | 1.364 | 0.112 | 0.614 | 0.187 |
| 464 | {BP_2Y,LDL_3Y,Smoking,Exercise} | {BMI_2Y} | 0.096 | 0.836 | 1.361 | 0.115 | 0.614 | 0.179 |
| 465 | {BP_2Y,FBG_A1c_3Y,Smoking,Exercise} | {BMI_2Y} | 0.096 | 0.832 | 1.355 | 0.116 | 0.614 | 0.159 |
| 466 | {BP_2Y,LDL_3Y,FBG_A1c_3Y,Exercise} | {BMI_2Y} | 0.096 | 0.836 | 1.362 | 0.115 | 0.614 | 0.181 |
| 467 | {BP_2Y,WC_2Y,LDL_3Y,FBG_A1c_3Y,Smoking} | {BMI_2Y} | 0.098 | 0.977 | 1.591 | 0.100 | 0.614 | 0.885 |
| 468 | {WC_2Y,LDL_3Y,FBG_A1c_3Y,Smoking} | {BMI_2Y} | 0.098 | 0.977 | 1.591 | 0.100 | 0.614 | 0.885 |
| 469 | {BP_2Y,LDL_3Y,Exercise} | {BMI_2Y} | 0.099 | 0.835 | 1.359 | 0.118 | 0.614 | 0.173 |
| 470 | {BP_2Y,FBG_A1c_3Y,Exercise} | {BMI_2Y} | 0.099 | 0.830 | 1.352 | 0.119 | 0.614 | 0.151 |
| 471 | {BP_2Y,WC_2Y,LDL_3Y,Smoking} | {BMI_2Y} | 0.100 | 0.977 | 1.592 | 0.102 | 0.614 | 0.887 |
| 472 | {WC_2Y,LDL_3Y,Smoking} | {BMI_2Y} | 0.100 | 0.977 | 1.592 | 0.103 | 0.614 | 0.887 |
| 473 | {BP_2Y,WC_2Y,FBG_A1c_3Y,Smoking} | {BMI_2Y} | 0.100 | 0.977 | 1.591 | 0.103 | 0.614 | 0.884 |
| 474 | {WC_2Y,FBG_A1c_3Y,Smoking} | {BMI_2Y} | 0.101 | 0.977 | 1.591 | 0.103 | 0.614 | 0.884 |
| 475 | {BP_2Y,Smoking,Alcohol,Exercise} | {BMI_2Y} | 0.101 | 0.815 | 1.328 | 0.123 | 0.614 | 0.076 |
| 476 | {BP_2Y,Alcohol,Exercise} | {BMI_2Y} | 0.102 | 0.814 | 1.326 | 0.126 | 0.614 | 0.071 |
| 477 | {BP_2Y,Smoking,Exercise} | {BMI_2Y} | 0.106 | 0.809 | 1.318 | 0.131 | 0.614 | 0.045 |
| 478 | {BP_2Y,WC_2Y,LDL_3Y,FBG_A1c_3Y} | {BMI_2Y} | 0.107 | 0.974 | 1.587 | 0.110 | 0.614 | 0.871 |
| 479 | {WC_2Y,LDL_3Y,FBG_A1c_3Y} | {BMI_2Y} | 0.107 | 0.974 | 1.587 | 0.110 | 0.614 | 0.871 |
| 480 | {BP_2Y,WC_2Y,LDL_3Y} | {BMI_2Y} | 0.109 | 0.975 | 1.588 | 0.112 | 0.614 | 0.874 |
| 481 | {BP_2Y,WC_2Y,Smoking} | {BMI_2Y} | 0.109 | 0.977 | 1.591 | 0.112 | 0.614 | 0.886 |
| 482 | {BP_2Y,Exercise} | {BMI_2Y} | 0.110 | 0.806 | 1.313 | 0.136 | 0.614 | 0.031 |
| 483 | {WC_2Y,Smoking} | {BMI_2Y} | 0.110 | 0.977 | 1.591 | 0.112 | 0.614 | 0.886 |
| 484 | {WC_2Y,LDL_3Y} | {BMI_2Y} | 0.110 | 0.975 | 1.588 | 0.112 | 0.614 | 0.874 |
| 485 | {BP_2Y,WC_2Y,FBG_A1c_3Y} | {BMI_2Y} | 0.110 | 0.974 | 1.586 | 0.113 | 0.614 | 0.870 |
| 486 | {WC_2Y,FBG_A1c_3Y} | {BMI_2Y} | 0.110 | 0.974 | 1.586 | 0.113 | 0.614 | 0.870 |
| 487 | {BP_2Y,WC_2Y} | {BMI_2Y} | 0.120 | 0.974 | 1.587 | 0.123 | 0.614 | 0.872 |
| 488 | {WC_2Y} | {BMI_2Y} | 0.120 | 0.975 | 1.587 | 0.123 | 0.614 | 0.873 |
| 489 | {BP_2Y,LDL_3Y,FBG_A1c_3Y,Smoking,Alcohol} | {BMI_2Y} | 0.345 | 0.812 | 1.323 | 0.424 | 0.614 | 0.061 |
| 490 | {BP_2Y,LDL_3Y,FBG_A1c_3Y,Alcohol} | {BMI_2Y} | 0.351 | 0.811 | 1.321 | 0.432 | 0.614 | 0.056 |
| 491 | {BP_2Y,LDL_3Y,Smoking,Alcohol} | {BMI_2Y} | 0.353 | 0.809 | 1.317 | 0.437 | 0.614 | 0.044 |
| 492 | {BP_2Y,FBG_A1c_3Y,Smoking,Alcohol} | {BMI_2Y} | 0.355 | 0.806 | 1.312 | 0.441 | 0.614 | 0.029 |
| 493 | {BP_2Y,LDL_3Y,Alcohol} | {BMI_2Y} | 0.360 | 0.808 | 1.316 | 0.445 | 0.614 | 0.039 |
| 494 | {BP_2Y,FBG_A1c_3Y,Alcohol} | {BMI_2Y} | 0.362 | 0.805 | 1.311 | 0.450 | 0.614 | 0.023 |
| 495 | {FBG_A1c_3Y,Diet,Exercise} | {Smoking} | 0.020 | 0.980 | 1.219 | 0.021 | 0.804 | 0.248 |
| 496 | {BP_2Y,Alcohol,Diet,Exercise} | {Smoking} | 0.021 | 0.982 | 1.221 | 0.021 | 0.804 | 0.619 |
| 497 | {BP_2Y,Diet,Exercise} | {Smoking} | 0.021 | 0.978 | 1.216 | 0.022 | 0.804 | 0.706 |
| 498 | {BP_2Y,BMI_2Y,WC_2Y,Exercise} | {Smoking} | 0.021 | 0.969 | 1.205 | 0.022 | 0.804 | 0.656 |
| 499 | {BMI_2Y,WC_2Y,Exercise} | {Smoking} | 0.021 | 0.969 | 1.205 | 0.022 | 0.804 | 0.660 |
| 500 | {BP_2Y,WC_2Y,Exercise} | {Smoking} | 0.022 | 0.969 | 1.204 | 0.022 | 0.804 | 0.688 |
| 501 | {WC_2Y,Exercise} | {Smoking} | 0.022 | 0.969 | 1.204 | 0.022 | 0.804 | 0.691 |
| 502 | {Alcohol,Diet,Exercise} | {Smoking} | 0.023 | 0.981 | 1.220 | 0.023 | 0.804 | 0.873 |
| 503 | {BP_2Y,BMI_2Y,LDL_3Y,FBG_A1c_3Y,Alcohol,Diet} | {Smoking} | 0.023 | 0.987 | 1.227 | 0.023 | 0.804 | 0.909 |
| 504 | {BMI_2Y,LDL_3Y,FBG_A1c_3Y,Alcohol,Diet} | {Smoking} | 0.023 | 0.987 | 1.226 | 0.023 | 0.804 | 0.909 |
| 505 | {BP_2Y,BMI_2Y,LDL_3Y,Alcohol,Diet} | {Smoking} | 0.023 | 0.987 | 1.226 | 0.024 | 0.804 | 0.912 |
| 506 | {BMI_2Y,LDL_3Y,Alcohol,Diet} | {Smoking} | 0.023 | 0.986 | 1.226 | 0.024 | 0.804 | 0.913 |
| 507 | {Diet,Exercise} | {Smoking} | 0.024 | 0.977 | 1.215 | 0.024 | 0.804 | 0.868 |
| 508 | {BP_2Y,BMI_2Y,FBG_A1c_3Y,Alcohol,Diet} | {Smoking} | 0.024 | 0.986 | 1.226 | 0.024 | 0.804 | 0.919 |
| 509 | {BP_2Y,BMI_2Y,LDL_3Y,FBG_A1c_3Y,Diet} | {Smoking} | 0.024 | 0.980 | 1.218 | 0.024 | 0.804 | 0.887 |
| 510 | {BMI_2Y,FBG_A1c_3Y,Alcohol,Diet} | {Smoking} | 0.024 | 0.986 | 1.225 | 0.024 | 0.804 | 0.919 |
| 511 | {BMI_2Y,LDL_3Y,FBG_A1c_3Y,Diet} | {Smoking} | 0.024 | 0.979 | 1.217 | 0.025 | 0.804 | 0.886 |
| 512 | {BP_2Y,BMI_2Y,LDL_3Y,Diet} | {Smoking} | 0.024 | 0.978 | 1.216 | 0.025 | 0.804 | 0.887 |
| 513 | {BMI_2Y,LDL_3Y,Diet} | {Smoking} | 0.024 | 0.978 | 1.215 | 0.025 | 0.804 | 0.886 |
| 514 | {BP_2Y,BMI_2Y,FBG_A1c_3Y,Diet} | {Smoking} | 0.025 | 0.979 | 1.217 | 0.025 | 0.804 | 0.895 |
| 515 | {BMI_2Y,FBG_A1c_3Y,Diet} | {Smoking} | 0.025 | 0.978 | 1.216 | 0.025 | 0.804 | 0.891 |
| 516 | {BP_2Y,LDL_3Y,FBG_A1c_3Y,Alcohol,Diet} | {Smoking} | 0.026 | 0.986 | 1.226 | 0.026 | 0.804 | 0.932 |
| 517 | {BP_2Y,BMI_2Y,Alcohol,Diet} | {Smoking} | 0.026 | 0.984 | 1.224 | 0.026 | 0.804 | 0.922 |
| 518 | {BMI_2Y,Alcohol,Diet} | {Smoking} | 0.026 | 0.984 | 1.223 | 0.027 | 0.804 | 0.918 |
| 519 | {BP_2Y,LDL_3Y,Alcohol,Diet} | {Smoking} | 0.026 | 0.986 | 1.225 | 0.027 | 0.804 | 0.928 |
| 520 | {BP_2Y,FBG_A1c_3Y,Alcohol,Diet} | {Smoking} | 0.027 | 0.986 | 1.225 | 0.027 | 0.804 | 0.928 |
| 521 | {BP_2Y,LDL_3Y,FBG_A1c_3Y,Diet} | {Smoking} | 0.027 | 0.976 | 1.213 | 0.028 | 0.804 | 0.878 |
| 522 | {BP_2Y,BMI_2Y,Diet} | {Smoking} | 0.027 | 0.977 | 1.214 | 0.028 | 0.804 | 0.884 |
| 523 | {LDL_3Y,FBG_A1c_3Y,Alcohol,Diet} | {Smoking} | 0.027 | 0.986 | 1.226 | 0.027 | 0.804 | 0.931 |
| 524 | {BMI_2Y,Diet} | {Smoking} | 0.027 | 0.976 | 1.213 | 0.028 | 0.804 | 0.879 |
| 525 | {BP_2Y,LDL_3Y,Diet} | {Smoking} | 0.027 | 0.974 | 1.211 | 0.028 | 0.804 | 0.872 |
| 526 | {LDL_3Y,Alcohol,Diet} | {Smoking} | 0.027 | 0.986 | 1.225 | 0.028 | 0.804 | 0.928 |
| 527 | {BP_2Y,FBG_A1c_3Y,Diet} | {Smoking} | 0.028 | 0.975 | 1.212 | 0.029 | 0.804 | 0.875 |
| 528 | {LDL_3Y,FBG_A1c_3Y,Diet} | {Smoking} | 0.028 | 0.975 | 1.212 | 0.029 | 0.804 | 0.874 |
| 529 | {FBG_A1c_3Y,Alcohol,Diet} | {Smoking} | 0.028 | 0.985 | 1.225 | 0.029 | 0.804 | 0.926 |
| 530 | {LDL_3Y,Diet} | {Smoking} | 0.029 | 0.974 | 1.210 | 0.029 | 0.804 | 0.869 |
| 531 | {FBG_A1c_3Y,Diet} | {Smoking} | 0.029 | 0.974 | 1.210 | 0.030 | 0.804 | 0.869 |
| 532 | {BP_2Y,Alcohol,Diet} | {Smoking} | 0.030 | 0.983 | 1.221 | 0.030 | 0.804 | 0.913 |
| 533 | {BP_2Y,Diet} | {Smoking} | 0.031 | 0.972 | 1.208 | 0.032 | 0.804 | 0.858 |
| 534 | {Alcohol,Diet} | {Smoking} | 0.034 | 0.982 | 1.220 | 0.034 | 0.804 | 0.908 |
| 535 | {Diet} | {Smoking} | 0.035 | 0.970 | 1.206 | 0.036 | 0.804 | 0.852 |
| 536 | {BP_2Y,BMI_2Y,WC_2Y,LDL_3Y,FBG_A1c_3Y,Alcohol} | {Smoking} | 0.080 | 0.987 | 1.227 | 0.082 | 0.804 | 0.934 |
| 537 | {BMI_2Y,WC_2Y,LDL_3Y,FBG_A1c_3Y,Alcohol} | {Smoking} | 0.080 | 0.987 | 1.227 | 0.082 | 0.804 | 0.933 |
| 538 | {BP_2Y,WC_2Y,LDL_3Y,FBG_A1c_3Y,Alcohol} | {Smoking} | 0.082 | 0.987 | 1.227 | 0.083 | 0.804 | 0.934 |
| 539 | {WC_2Y,LDL_3Y,FBG_A1c_3Y,Alcohol} | {Smoking} | 0.082 | 0.987 | 1.227 | 0.083 | 0.804 | 0.934 |
| 540 | {BP_2Y,BMI_2Y,WC_2Y,LDL_3Y,Alcohol} | {Smoking} | 0.082 | 0.987 | 1.227 | 0.083 | 0.804 | 0.935 |
| 541 | {BMI_2Y,WC_2Y,LDL_3Y,Alcohol} | {Smoking} | 0.082 | 0.987 | 1.227 | 0.083 | 0.804 | 0.934 |
| 542 | {BP_2Y,BMI_2Y,WC_2Y,FBG_A1c_3Y,Alcohol} | {Smoking} | 0.082 | 0.987 | 1.227 | 0.084 | 0.804 | 0.934 |
| 543 | {BMI_2Y,WC_2Y,FBG_A1c_3Y,Alcohol} | {Smoking} | 0.082 | 0.987 | 1.226 | 0.084 | 0.804 | 0.933 |
| 544 | {BP_2Y,WC_2Y,LDL_3Y,Alcohol} | {Smoking} | 0.083 | 0.987 | 1.227 | 0.085 | 0.804 | 0.935 |
| 545 | {WC_2Y,LDL_3Y,Alcohol} | {Smoking} | 0.083 | 0.987 | 1.227 | 0.085 | 0.804 | 0.935 |
| 546 | {BP_2Y,WC_2Y,FBG_A1c_3Y,Alcohol} | {Smoking} | 0.084 | 0.987 | 1.227 | 0.085 | 0.804 | 0.934 |
| 547 | {WC_2Y,FBG_A1c_3Y,Alcohol} | {Smoking} | 0.084 | 0.987 | 1.226 | 0.085 | 0.804 | 0.933 |
| 548 | {BP_2Y,BMI_2Y,LDL_3Y,FBG_A1c_3Y,Alcohol,Exercise} | {Smoking} | 0.089 | 0.984 | 1.223 | 0.090 | 0.804 | 0.918 |
| 549 | {BMI_2Y,LDL_3Y,FBG_A1c_3Y,Alcohol,Exercise} | {Smoking} | 0.090 | 0.983 | 1.222 | 0.091 | 0.804 | 0.917 |
| 550 | {BP_2Y,BMI_2Y,WC_2Y,Alcohol} | {Smoking} | 0.091 | 0.986 | 1.226 | 0.092 | 0.804 | 0.931 |
| 551 | {BMI_2Y,WC_2Y,Alcohol} | {Smoking} | 0.091 | 0.986 | 1.226 | 0.092 | 0.804 | 0.930 |
| 552 | {BP_2Y,BMI_2Y,LDL_3Y,Alcohol,Exercise} | {Smoking} | 0.091 | 0.983 | 1.222 | 0.093 | 0.804 | 0.916 |
| 553 | {BP_2Y,BMI_2Y,FBG_A1c_3Y,Alcohol,Exercise} | {Smoking} | 0.091 | 0.983 | 1.222 | 0.093 | 0.804 | 0.916 |
| 554 | {BP_2Y,WC_2Y,Alcohol} | {Smoking} | 0.092 | 0.986 | 1.226 | 0.093 | 0.804 | 0.931 |
| 555 | {BMI_2Y,LDL_3Y,Alcohol,Exercise} | {Smoking} | 0.092 | 0.983 | 1.222 | 0.093 | 0.804 | 0.915 |
| 556 | {WC_2Y,Alcohol} | {Smoking} | 0.092 | 0.986 | 1.226 | 0.093 | 0.804 | 0.931 |
| 557 | {BMI_2Y,FBG_A1c_3Y,Alcohol,Exercise} | {Smoking} | 0.092 | 0.983 | 1.222 | 0.094 | 0.804 | 0.915 |
| 558 | {BP_2Y,BMI_2Y,LDL_3Y,FBG_A1c_3Y,Exercise} | {Smoking} | 0.094 | 0.972 | 1.209 | 0.096 | 0.804 | 0.862 |
| 559 | {BMI_2Y,LDL_3Y,FBG_A1c_3Y,Exercise} | {Smoking} | 0.095 | 0.972 | 1.209 | 0.097 | 0.804 | 0.862 |
| 560 | {BP_2Y,BMI_2Y,LDL_3Y,Exercise} | {Smoking} | 0.096 | 0.972 | 1.208 | 0.099 | 0.804 | 0.859 |
| 561 | {BP_2Y,BMI_2Y,FBG_A1c_3Y,Exercise} | {Smoking} | 0.096 | 0.972 | 1.208 | 0.099 | 0.804 | 0.859 |
| 562 | {BMI_2Y,LDL_3Y,Exercise} | {Smoking} | 0.097 | 0.972 | 1.208 | 0.100 | 0.804 | 0.859 |
| 563 | {BMI_2Y,FBG_A1c_3Y,Exercise} | {Smoking} | 0.097 | 0.972 | 1.208 | 0.100 | 0.804 | 0.858 |
| 564 | {BP_2Y,BMI_2Y,WC_2Y,LDL_3Y,FBG_A1c_3Y} | {Smoking} | 0.098 | 0.914 | 1.137 | 0.107 | 0.804 | 0.572 |
| 565 | {BMI_2Y,WC_2Y,LDL_3Y,FBG_A1c_3Y} | {Smoking} | 0.098 | 0.914 | 1.136 | 0.107 | 0.804 | 0.571 |
| 566 | {BP_2Y,BMI_2Y,WC_2Y,LDL_3Y} | {Smoking} | 0.100 | 0.915 | 1.137 | 0.109 | 0.804 | 0.575 |
| 567 | {BMI_2Y,WC_2Y,LDL_3Y} | {Smoking} | 0.100 | 0.915 | 1.137 | 0.110 | 0.804 | 0.573 |
| 568 | {BP_2Y,WC_2Y,LDL_3Y,FBG_A1c_3Y} | {Smoking} | 0.100 | 0.912 | 1.134 | 0.110 | 0.804 | 0.560 |
| 569 | {WC_2Y,LDL_3Y,FBG_A1c_3Y} | {Smoking} | 0.100 | 0.912 | 1.133 | 0.110 | 0.804 | 0.558 |
| 570 | {BP_2Y,BMI_2Y,WC_2Y,FBG_A1c_3Y} | {Smoking} | 0.100 | 0.914 | 1.136 | 0.110 | 0.804 | 0.571 |
| 571 | {BMI_2Y,WC_2Y,FBG_A1c_3Y} | {Smoking} | 0.101 | 0.914 | 1.136 | 0.110 | 0.804 | 0.570 |
| 572 | {BP_2Y,BMI_2Y,Alcohol,Exercise} | {Smoking} | 0.101 | 0.982 | 1.221 | 0.102 | 0.804 | 0.910 |
| 573 | {BMI_2Y,Alcohol,Exercise} | {Smoking} | 0.102 | 0.982 | 1.220 | 0.103 | 0.804 | 0.909 |
| 574 | {BP_2Y,WC_2Y,LDL_3Y} | {Smoking} | 0.102 | 0.912 | 1.134 | 0.112 | 0.804 | 0.562 |
| 575 | {WC_2Y,LDL_3Y} | {Smoking} | 0.103 | 0.912 | 1.134 | 0.112 | 0.804 | 0.561 |
| 576 | {BP_2Y,WC_2Y,FBG_A1c_3Y} | {Smoking} | 0.103 | 0.912 | 1.133 | 0.113 | 0.804 | 0.558 |
| 577 | {WC_2Y,FBG_A1c_3Y} | {Smoking} | 0.103 | 0.911 | 1.133 | 0.113 | 0.804 | 0.557 |
| 578 | {BP_2Y,LDL_3Y,FBG_A1c_3Y,Alcohol,Exercise} | {Smoking} | 0.106 | 0.983 | 1.222 | 0.107 | 0.804 | 0.916 |
| 579 | {BP_2Y,BMI_2Y,Exercise} | {Smoking} | 0.106 | 0.970 | 1.205 | 0.110 | 0.804 | 0.848 |
| 580 | {BMI_2Y,Exercise} | {Smoking} | 0.107 | 0.970 | 1.205 | 0.111 | 0.804 | 0.848 |
| 581 | {BP_2Y,LDL_3Y,Alcohol,Exercise} | {Smoking} | 0.108 | 0.983 | 1.222 | 0.110 | 0.804 | 0.914 |
| 582 | {BP_2Y,FBG_A1c_3Y,Alcohol,Exercise} | {Smoking} | 0.109 | 0.983 | 1.222 | 0.111 | 0.804 | 0.914 |
| 583 | {BP_2Y,BMI_2Y,WC_2Y} | {Smoking} | 0.109 | 0.913 | 1.135 | 0.120 | 0.804 | 0.566 |
| 584 | {BMI_2Y,WC_2Y} | {Smoking} | 0.110 | 0.913 | 1.135 | 0.120 | 0.804 | 0.564 |
| 585 | {BP_2Y,WC_2Y} | {Smoking} | 0.112 | 0.911 | 1.132 | 0.123 | 0.804 | 0.554 |
| 586 | {BP_2Y,LDL_3Y,FBG_A1c_3Y,Exercise} | {Smoking} | 0.112 | 0.971 | 1.207 | 0.115 | 0.804 | 0.856 |
| 587 | {WC_2Y} | {Smoking} | 0.112 | 0.910 | 1.132 | 0.123 | 0.804 | 0.552 |
| 588 | {LDL_3Y,FBG_A1c_3Y,Alcohol,Exercise} | {Smoking} | 0.113 | 0.983 | 1.222 | 0.115 | 0.804 | 0.915 |
| 589 | {BP_2Y,LDL_3Y,Exercise} | {Smoking} | 0.115 | 0.970 | 1.206 | 0.118 | 0.804 | 0.852 |
| 590 | {BP_2Y,FBG_A1c_3Y,Exercise} | {Smoking} | 0.116 | 0.970 | 1.206 | 0.119 | 0.804 | 0.849 |
| 591 | {LDL_3Y,Alcohol,Exercise} | {Smoking} | 0.117 | 0.983 | 1.221 | 0.119 | 0.804 | 0.913 |
| 592 | {FBG_A1c_3Y,Alcohol,Exercise} | {Smoking} | 0.117 | 0.982 | 1.221 | 0.120 | 0.804 | 0.912 |
| 593 | {LDL_3Y,FBG_A1c_3Y,Exercise} | {Smoking} | 0.120 | 0.971 | 1.207 | 0.124 | 0.804 | 0.854 |
| 594 | {BP_2Y,Alcohol,Exercise} | {Smoking} | 0.123 | 0.981 | 1.219 | 0.126 | 0.804 | 0.903 |
| 595 | {LDL_3Y,Exercise} | {Smoking} | 0.124 | 0.970 | 1.206 | 0.127 | 0.804 | 0.850 |
| 596 | {FBG_A1c_3Y,Exercise} | {Smoking} | 0.125 | 0.969 | 1.205 | 0.129 | 0.804 | 0.847 |
| 597 | {BP_2Y,Exercise} | {Smoking} | 0.131 | 0.966 | 1.201 | 0.136 | 0.804 | 0.831 |
| 598 | {Alcohol,Exercise} | {Smoking} | 0.139 | 0.980 | 1.218 | 0.142 | 0.804 | 0.900 |
| 599 | {Exercise} | {Smoking} | 0.148 | 0.965 | 1.200 | 0.153 | 0.804 | 0.827 |
| 600 | {BP_2Y,BMI_2Y,LDL_3Y,FBG_A1c_3Y,Alcohol} | {Smoking} | 0.345 | 0.983 | 1.221 | 0.351 | 0.804 | 0.913 |
| 601 | {BMI_2Y,LDL_3Y,FBG_A1c_3Y,Alcohol} | {Smoking} | 0.348 | 0.983 | 1.221 | 0.355 | 0.804 | 0.913 |
| 602 | {BP_2Y,BMI_2Y,LDL_3Y,Alcohol} | {Smoking} | 0.353 | 0.982 | 1.221 | 0.360 | 0.804 | 0.911 |
| 603 | {BP_2Y,BMI_2Y,FBG_A1c_3Y,Alcohol} | {Smoking} | 0.355 | 0.982 | 1.221 | 0.362 | 0.804 | 0.909 |
| 604 | {BMI_2Y,LDL_3Y,Alcohol} | {Smoking} | 0.357 | 0.982 | 1.221 | 0.364 | 0.804 | 0.911 |
| 605 | {BMI_2Y,FBG_A1c_3Y,Alcohol} | {Smoking} | 0.360 | 0.982 | 1.221 | 0.366 | 0.804 | 0.909 |
| 606 | {BP_2Y,BMI_2Y,Alcohol} | {Smoking} | 0.394 | 0.981 | 1.219 | 0.401 | 0.804 | 0.903 |
| 607 | {BMI_2Y,Alcohol} | {Smoking} | 0.399 | 0.981 | 1.219 | 0.407 | 0.804 | 0.903 |
| 608 | {BP_2Y,LDL_3Y,FBG_A1c_3Y,Alcohol} | {Smoking} | 0.424 | 0.981 | 1.220 | 0.432 | 0.804 | 0.907 |
| 609 | {BP_2Y,LDL_3Y,Alcohol} | {Smoking} | 0.437 | 0.981 | 1.220 | 0.445 | 0.804 | 0.905 |
| 610 | {BP_2Y,FBG_A1c_3Y,Alcohol} | {Smoking} | 0.441 | 0.981 | 1.219 | 0.450 | 0.804 | 0.903 |
| 611 | {BP_2Y,BMI_2Y,LDL_3Y,FBG_A1c_3Y} | {Smoking} | 0.456 | 0.876 | 1.089 | 0.520 | 0.804 | 0.380 |
| 612 | {LDL_3Y,FBG_A1c_3Y,Alcohol} | {Smoking} | 0.458 | 0.981 | 1.219 | 0.467 | 0.804 | 0.904 |
| 613 | {BMI_2Y,LDL_3Y,FBG_A1c_3Y} | {Smoking} | 0.461 | 0.875 | 1.088 | 0.526 | 0.804 | 0.377 |
| 614 | {BP_2Y,BMI_2Y,LDL_3Y} | {Smoking} | 0.467 | 0.874 | 1.086 | 0.535 | 0.804 | 0.370 |
| 615 | {BP_2Y,BMI_2Y,FBG_A1c_3Y} | {Smoking} | 0.471 | 0.873 | 1.086 | 0.540 | 0.804 | 0.367 |
| 616 | {LDL_3Y,Alcohol} | {Smoking} | 0.473 | 0.980 | 1.219 | 0.482 | 0.804 | 0.902 |
| 617 | {BMI_2Y,LDL_3Y} | {Smoking} | 0.473 | 0.873 | 1.086 | 0.541 | 0.804 | 0.367 |
| 618 | {BMI_2Y,FBG_A1c_3Y} | {Smoking} | 0.477 | 0.873 | 1.085 | 0.547 | 0.804 | 0.364 |
| 619 | {FBG_A1c_3Y,Alcohol} | {Smoking} | 0.479 | 0.980 | 1.218 | 0.489 | 0.804 | 0.899 |
| 620 | {BP_2Y,Alcohol} | {Smoking} | 0.505 | 0.979 | 1.217 | 0.516 | 0.804 | 0.894 |
| 621 | {BP_2Y,BMI_2Y} | {Smoking} | 0.524 | 0.867 | 1.077 | 0.605 | 0.804 | 0.334 |
| 622 | {BMI_2Y} | {Smoking} | 0.532 | 0.866 | 1.076 | 0.614 | 0.804 | 0.329 |
| 623 | {BP_2Y,LDL_3Y,FBG_A1c_3Y} | {Smoking} | 0.572 | 0.860 | 1.069 | 0.666 | 0.804 | 0.299 |
| 624 | {Alcohol} | {Smoking} | 0.576 | 0.977 | 1.215 | 0.589 | 0.804 | 0.887 |
| 625 | {BP_2Y,LDL_3Y} | {Smoking} | 0.589 | 0.858 | 1.066 | 0.687 | 0.804 | 0.288 |
| 626 | {BP_2Y,FBG_A1c_3Y} | {Smoking} | 0.598 | 0.854 | 1.062 | 0.700 | 0.804 | 0.271 |
| 627 | {LDL_3Y,FBG_A1c_3Y} | {Smoking} | 0.628 | 0.847 | 1.053 | 0.741 | 0.804 | 0.235 |
| 628 | {LDL_3Y} | {Smoking} | 0.648 | 0.844 | 1.049 | 0.767 | 0.804 | 0.221 |
| 629 | {FBG_A1c_3Y} | {Smoking} | 0.662 | 0.840 | 1.044 | 0.788 | 0.804 | 0.200 |
| 630 | {BP_2Y} | {Smoking} | 0.689 | 0.840 | 1.044 | 0.820 | 0.804 | 0.220 |
| 631 | {BP_2Y,Smoking,Diet,Exercise} | {Alcohol} | 0.021 | 0.975 | 1.656 | 0.021 | 0.589 | 0.535 |
| 632 | {BP_2Y,Diet,Exercise} | {Alcohol} | 0.021 | 0.971 | 1.649 | 0.022 | 0.589 | 0.610 |
| 633 | {Smoking,Diet,Exercise} | {Alcohol} | 0.023 | 0.975 | 1.656 | 0.024 | 0.589 | 0.836 |
| 634 | {BP_2Y,BMI_2Y,LDL_3Y,FBG_A1c_3Y,Smoking,Diet} | {Alcohol} | 0.023 | 0.962 | 1.633 | 0.024 | 0.589 | 0.768 |
| 635 | {BMI_2Y,LDL_3Y,FBG_A1c_3Y,Smoking,Diet} | {Alcohol} | 0.023 | 0.962 | 1.634 | 0.024 | 0.589 | 0.776 |
| 636 | {BP_2Y,BMI_2Y,LDL_3Y,Smoking,Diet} | {Alcohol} | 0.023 | 0.962 | 1.634 | 0.024 | 0.589 | 0.782 |
| 637 | {BP_2Y,BMI_2Y,LDL_3Y,FBG_A1c_3Y,Diet} | {Alcohol} | 0.023 | 0.955 | 1.621 | 0.024 | 0.589 | 0.750 |
| 638 | {BMI_2Y,LDL_3Y,Smoking,Diet} | {Alcohol} | 0.023 | 0.963 | 1.634 | 0.024 | 0.589 | 0.789 |
| 639 | {Diet,Exercise} | {Alcohol} | 0.023 | 0.971 | 1.649 | 0.024 | 0.589 | 0.831 |
| 640 | {BMI_2Y,LDL_3Y,FBG_A1c_3Y,Diet} | {Alcohol} | 0.023 | 0.955 | 1.621 | 0.025 | 0.589 | 0.756 |
| 641 | {BP_2Y,BMI_2Y,LDL_3Y,Diet} | {Alcohol} | 0.024 | 0.954 | 1.621 | 0.025 | 0.589 | 0.760 |
| 642 | {BMI_2Y,LDL_3Y,Diet} | {Alcohol} | 0.024 | 0.954 | 1.620 | 0.025 | 0.589 | 0.766 |
| 643 | {BP_2Y,BMI_2Y,FBG_A1c_3Y,Smoking,Diet} | {Alcohol} | 0.024 | 0.962 | 1.634 | 0.025 | 0.589 | 0.803 |
| 644 | {BMI_2Y,FBG_A1c_3Y,Smoking,Diet} | {Alcohol} | 0.024 | 0.962 | 1.634 | 0.025 | 0.589 | 0.809 |
| 645 | {BP_2Y,BMI_2Y,FBG_A1c_3Y,Diet} | {Alcohol} | 0.024 | 0.955 | 1.622 | 0.025 | 0.589 | 0.777 |
| 646 | {BMI_2Y,FBG_A1c_3Y,Diet} | {Alcohol} | 0.024 | 0.955 | 1.622 | 0.025 | 0.589 | 0.776 |
| 647 | {BP_2Y,LDL_3Y,FBG_A1c_3Y,Smoking,Diet} | {Alcohol} | 0.026 | 0.960 | 1.631 | 0.027 | 0.589 | 0.802 |
| 648 | {BP_2Y,BMI_2Y,Smoking,Diet} | {Alcohol} | 0.026 | 0.963 | 1.635 | 0.027 | 0.589 | 0.815 |
| 649 | {BMI_2Y,Smoking,Diet} | {Alcohol} | 0.026 | 0.963 | 1.635 | 0.027 | 0.589 | 0.814 |
| 650 | {BP_2Y,LDL_3Y,FBG_A1c_3Y,Diet} | {Alcohol} | 0.026 | 0.950 | 1.613 | 0.028 | 0.589 | 0.750 |
| 651 | {BP_2Y,LDL_3Y,Smoking,Diet} | {Alcohol} | 0.026 | 0.961 | 1.631 | 0.027 | 0.589 | 0.803 |
| 652 | {BP_2Y,BMI_2Y,Diet} | {Alcohol} | 0.026 | 0.955 | 1.623 | 0.028 | 0.589 | 0.777 |
| 653 | {BP_2Y,LDL_3Y,Diet} | {Alcohol} | 0.027 | 0.950 | 1.612 | 0.028 | 0.589 | 0.748 |
| 654 | {BMI_2Y,Diet} | {Alcohol} | 0.027 | 0.955 | 1.622 | 0.028 | 0.589 | 0.775 |
| 655 | {BP_2Y,FBG_A1c_3Y,Smoking,Diet} | {Alcohol} | 0.027 | 0.961 | 1.631 | 0.028 | 0.589 | 0.803 |
| 656 | {LDL_3Y,FBG_A1c_3Y,Smoking,Diet} | {Alcohol} | 0.027 | 0.961 | 1.631 | 0.028 | 0.589 | 0.804 |
| 657 | {BP_2Y,FBG_A1c_3Y,Diet} | {Alcohol} | 0.027 | 0.950 | 1.614 | 0.029 | 0.589 | 0.751 |
| 658 | {LDL_3Y,FBG_A1c_3Y,Diet} | {Alcohol} | 0.027 | 0.950 | 1.613 | 0.029 | 0.589 | 0.748 |
| 659 | {LDL_3Y,Smoking,Diet} | {Alcohol} | 0.027 | 0.961 | 1.632 | 0.029 | 0.589 | 0.804 |
| 660 | {LDL_3Y,Diet} | {Alcohol} | 0.028 | 0.949 | 1.612 | 0.029 | 0.589 | 0.747 |
| 661 | {FBG_A1c_3Y,Smoking,Diet} | {Alcohol} | 0.028 | 0.961 | 1.632 | 0.029 | 0.589 | 0.806 |
| 662 | {FBG_A1c_3Y,Diet} | {Alcohol} | 0.029 | 0.950 | 1.613 | 0.030 | 0.589 | 0.750 |
| 663 | {BP_2Y,Smoking,Diet} | {Alcohol} | 0.030 | 0.960 | 1.631 | 0.031 | 0.589 | 0.802 |
| 664 | {BP_2Y,Diet} | {Alcohol} | 0.030 | 0.950 | 1.613 | 0.032 | 0.589 | 0.748 |
| 665 | {Smoking,Diet} | {Alcohol} | 0.034 | 0.961 | 1.632 | 0.035 | 0.589 | 0.804 |
| 666 | {Diet} | {Alcohol} | 0.034 | 0.950 | 1.613 | 0.036 | 0.589 | 0.750 |
| 667 | {BP_2Y,BMI_2Y,WC_2Y,LDL_3Y,FBG_A1c_3Y,Smoking} | {Alcohol} | 0.080 | 0.821 | 1.393 | 0.098 | 0.589 | 0.103 |
| 668 | {BMI_2Y,WC_2Y,LDL_3Y,FBG_A1c_3Y,Smoking} | {Alcohol} | 0.080 | 0.820 | 1.393 | 0.098 | 0.589 | 0.102 |
| 669 | {BP_2Y,WC_2Y,LDL_3Y,FBG_A1c_3Y,Smoking} | {Alcohol} | 0.082 | 0.813 | 1.380 | 0.100 | 0.589 | 0.063 |
| 670 | {WC_2Y,LDL_3Y,FBG_A1c_3Y,Smoking} | {Alcohol} | 0.082 | 0.812 | 1.379 | 0.100 | 0.589 | 0.062 |
| 671 | {BP_2Y,BMI_2Y,WC_2Y,LDL_3Y,Smoking} | {Alcohol} | 0.082 | 0.822 | 1.396 | 0.100 | 0.589 | 0.111 |
| 672 | {BMI_2Y,WC_2Y,LDL_3Y,Smoking} | {Alcohol} | 0.082 | 0.822 | 1.396 | 0.100 | 0.589 | 0.110 |
| 673 | {BP_2Y,BMI_2Y,WC_2Y,FBG_A1c_3Y,Smoking} | {Alcohol} | 0.082 | 0.821 | 1.394 | 0.100 | 0.589 | 0.103 |
| 674 | {BMI_2Y,WC_2Y,FBG_A1c_3Y,Smoking} | {Alcohol} | 0.082 | 0.820 | 1.393 | 0.101 | 0.589 | 0.102 |
| 675 | {BP_2Y,WC_2Y,LDL_3Y,Smoking} | {Alcohol} | 0.083 | 0.814 | 1.383 | 0.102 | 0.589 | 0.072 |
| 676 | {WC_2Y,LDL_3Y,Smoking} | {Alcohol} | 0.083 | 0.814 | 1.383 | 0.103 | 0.589 | 0.071 |
| 677 | {BP_2Y,WC_2Y,FBG_A1c_3Y,Smoking} | {Alcohol} | 0.084 | 0.813 | 1.380 | 0.103 | 0.589 | 0.063 |
| 678 | {WC_2Y,FBG_A1c_3Y,Smoking} | {Alcohol} | 0.084 | 0.812 | 1.380 | 0.103 | 0.589 | 0.062 |
| 679 | {BP_2Y,BMI_2Y,LDL_3Y,FBG_A1c_3Y,Smoking,Exercise} | {Alcohol} | 0.089 | 0.947 | 1.608 | 0.094 | 0.589 | 0.734 |
| 680 | {BMI_2Y,LDL_3Y,FBG_A1c_3Y,Smoking,Exercise} | {Alcohol} | 0.090 | 0.947 | 1.608 | 0.095 | 0.589 | 0.734 |
| 681 | {BP_2Y,BMI_2Y,LDL_3Y,FBG_A1c_3Y,Exercise} | {Alcohol} | 0.090 | 0.936 | 1.590 | 0.096 | 0.589 | 0.681 |
| 682 | {BP_2Y,BMI_2Y,WC_2Y,Smoking} | {Alcohol} | 0.091 | 0.827 | 1.405 | 0.109 | 0.589 | 0.137 |
| 683 | {BMI_2Y,WC_2Y,Smoking} | {Alcohol} | 0.091 | 0.827 | 1.405 | 0.110 | 0.589 | 0.136 |
| 684 | {BMI_2Y,LDL_3Y,FBG_A1c_3Y,Exercise} | {Alcohol} | 0.091 | 0.936 | 1.590 | 0.097 | 0.589 | 0.681 |
| 685 | {BP_2Y,BMI_2Y,LDL_3Y,Smoking,Exercise} | {Alcohol} | 0.091 | 0.948 | 1.609 | 0.096 | 0.589 | 0.739 |
| 686 | {BP_2Y,BMI_2Y,FBG_A1c_3Y,Smoking,Exercise} | {Alcohol} | 0.091 | 0.947 | 1.607 | 0.096 | 0.589 | 0.733 |
| 687 | {BP_2Y,WC_2Y,Smoking} | {Alcohol} | 0.092 | 0.819 | 1.391 | 0.112 | 0.589 | 0.096 |
| 688 | {BMI_2Y,LDL_3Y,Smoking,Exercise} | {Alcohol} | 0.092 | 0.948 | 1.609 | 0.097 | 0.589 | 0.738 |
| 689 | {WC_2Y,Smoking} | {Alcohol} | 0.092 | 0.819 | 1.391 | 0.112 | 0.589 | 0.095 |
| 690 | {BMI_2Y,FBG_A1c_3Y,Smoking,Exercise} | {Alcohol} | 0.092 | 0.947 | 1.607 | 0.097 | 0.589 | 0.733 |
| 691 | {BP_2Y,BMI_2Y,LDL_3Y,Exercise} | {Alcohol} | 0.093 | 0.937 | 1.591 | 0.099 | 0.589 | 0.684 |
| 692 | {BP_2Y,BMI_2Y,FBG_A1c_3Y,Exercise} | {Alcohol} | 0.093 | 0.935 | 1.589 | 0.099 | 0.589 | 0.677 |
| 693 | {BMI_2Y,LDL_3Y,Exercise} | {Alcohol} | 0.093 | 0.937 | 1.591 | 0.100 | 0.589 | 0.684 |
| 694 | {BMI_2Y,FBG_A1c_3Y,Exercise} | {Alcohol} | 0.094 | 0.936 | 1.589 | 0.100 | 0.589 | 0.678 |
| 695 | {BP_2Y,BMI_2Y,Smoking,Exercise} | {Alcohol} | 0.101 | 0.947 | 1.608 | 0.106 | 0.589 | 0.736 |
| 696 | {BMI_2Y,Smoking,Exercise} | {Alcohol} | 0.102 | 0.947 | 1.608 | 0.107 | 0.589 | 0.736 |
| 697 | {BP_2Y,BMI_2Y,Exercise} | {Alcohol} | 0.102 | 0.935 | 1.588 | 0.110 | 0.589 | 0.676 |
| 698 | {BMI_2Y,Exercise} | {Alcohol} | 0.103 | 0.935 | 1.588 | 0.111 | 0.589 | 0.677 |
| 699 | {BP_2Y,LDL_3Y,FBG_A1c_3Y,Smoking,Exercise} | {Alcohol} | 0.106 | 0.942 | 1.600 | 0.112 | 0.589 | 0.711 |
| 700 | {BP_2Y,LDL_3Y,FBG_A1c_3Y,Exercise} | {Alcohol} | 0.107 | 0.931 | 1.580 | 0.115 | 0.589 | 0.653 |
| 701 | {BP_2Y,LDL_3Y,Smoking,Exercise} | {Alcohol} | 0.108 | 0.943 | 1.601 | 0.115 | 0.589 | 0.715 |
| 702 | {BP_2Y,FBG_A1c_3Y,Smoking,Exercise} | {Alcohol} | 0.109 | 0.941 | 1.598 | 0.116 | 0.589 | 0.706 |
| 703 | {BP_2Y,LDL_3Y,Exercise} | {Alcohol} | 0.110 | 0.931 | 1.581 | 0.118 | 0.589 | 0.656 |
| 704 | {BP_2Y,FBG_A1c_3Y,Exercise} | {Alcohol} | 0.111 | 0.929 | 1.577 | 0.119 | 0.589 | 0.644 |
| 705 | {LDL_3Y,FBG_A1c_3Y,Smoking,Exercise} | {Alcohol} | 0.113 | 0.943 | 1.602 | 0.120 | 0.589 | 0.716 |
| 706 | {LDL_3Y,FBG_A1c_3Y,Exercise} | {Alcohol} | 0.115 | 0.932 | 1.582 | 0.124 | 0.589 | 0.658 |
| 707 | {LDL_3Y,Smoking,Exercise} | {Alcohol} | 0.117 | 0.944 | 1.603 | 0.124 | 0.589 | 0.720 |
| 708 | {FBG_A1c_3Y,Smoking,Exercise} | {Alcohol} | 0.117 | 0.942 | 1.600 | 0.125 | 0.589 | 0.710 |
| 709 | {LDL_3Y,Exercise} | {Alcohol} | 0.119 | 0.932 | 1.583 | 0.127 | 0.589 | 0.660 |
| 710 | {FBG_A1c_3Y,Exercise} | {Alcohol} | 0.120 | 0.930 | 1.579 | 0.129 | 0.589 | 0.648 |
| 711 | {BP_2Y,Smoking,Exercise} | {Alcohol} | 0.123 | 0.940 | 1.596 | 0.131 | 0.589 | 0.700 |
| 712 | {BP_2Y,Exercise} | {Alcohol} | 0.126 | 0.926 | 1.573 | 0.136 | 0.589 | 0.631 |
| 713 | {Smoking,Exercise} | {Alcohol} | 0.139 | 0.941 | 1.598 | 0.148 | 0.589 | 0.704 |
| 714 | {Exercise} | {Alcohol} | 0.142 | 0.927 | 1.574 | 0.153 | 0.589 | 0.634 |

**S2 Table. Details on support, confidence, lift (raw and standardized) for 33-pruned association rules stratified with respect to consequent CDPSM element.**

| Rule number | Antecedent | Consequent | Support | Confidence | Lift | Support for Antecedent | Support for Consequent | Standardized lift |
| --- | --- | --- | --- | --- | --- | --- | --- | --- |
| 1 | {WC_2Y,LDL_3Y,FBG_A1c_3Y,Exercise} | {BP_2Y} | 0.020 | 0.999 | 1.219 | 0.020 | 0.820 | 0.999 |
| 2 | {BMI_2Y,WC_2Y,FBG_A1c_3Y,Exercise} | {BP_2Y} | 0.020 | 0.999 | 1.219 | 0.020 | 0.820 | 0.999 |
| 3 | {Smoking,Alcohol,Diet,Exercise} | {BP_2Y} | 0.021 | 0.896 | 1.093 | 0.023 | 0.820 | 0.896 |
| 4 | {BMI_2Y,WC_2Y,Smoking,Exercise} | {BP_2Y} | 0.021 | 0.999 | 1.218 | 0.021 | 0.820 | 0.999 |
| 5 | {BMI_2Y,LDL_3Y,FBG_A1c_3Y,Smoking,Alcohol,Diet} | {BP_2Y} | 0.023 | 0.994 | 1.213 | 0.023 | 0.820 | 0.994 |
| 6 | {BMI_2Y,WC_2Y,LDL_3Y,FBG_A1c_3Y,Smoking,Alcohol} | {BP_2Y} | 0.080 | 0.999 | 1.219 | 0.080 | 0.820 | 0.999 |
| 7 | {BMI_2Y,LDL_3Y,FBG_A1c_3Y,Smoking,Alcohol,Exercise} | {BP_2Y} | 0.089 | 0.992 | 1.209 | 0.090 | 0.820 | 0.992 |
| 8 | {BP_2Y,WC_2Y,FBG_A1c_3Y,Exercise} | {LDL_3Y} | 0.020 | 0.985 | 1.283 | 0.020 | 0.767 | 0.985 |
| 9 | {Diet,Exercise} | {LDL_3Y} | 0.020 | 0.830 | 1.082 | 0.024 | 0.767 | 0.830 |
| 10 | {BP_2Y,BMI_2Y,FBG_A1c_3Y,Smoking,Alcohol,Diet} | {LDL_3Y} | 0.023 | 0.967 | 1.261 | 0.024 | 0.767 | 0.967 |
| 11 | {BP_2Y,BMI_2Y,WC_2Y,FBG_A1c_3Y,Smoking,Alcohol} | {LDL_3Y} | 0.080 | 0.976 | 1.272 | 0.082 | 0.767 | 0.976 |
| 12 | {BP_2Y,BMI_2Y,FBG_A1c_3Y,Smoking,Alcohol,Exercise} | {LDL_3Y} | 0.089 | 0.974 | 1.270 | 0.091 | 0.767 | 0.974 |
| 13 | {BP_2Y,WC_2Y,LDL_3Y,Exercise} | {FBG_A1c_3Y} | 0.020 | 0.990 | 1.256 | 0.020 | 0.788 | 0.990 |
| 14 | {BP_2Y,BMI_2Y,WC_2Y,Exercise} | {FBG_A1c_3Y} | 0.020 | 0.914 | 1.160 | 0.022 | 0.788 | 0.914 |
| 15 | {Smoking,Diet,Exercise} | {FBG_A1c_3Y} | 0.020 | 0.854 | 1.084 | 0.024 | 0.788 | 0.854 |
| 16 | {BP_2Y,BMI_2Y,LDL_3Y,Smoking,Alcohol,Diet} | {FBG_A1c_3Y} | 0.023 | 0.989 | 1.256 | 0.023 | 0.788 | 0.989 |
| 17 | {BP_2Y,BMI_2Y,WC_2Y,LDL_3Y,Smoking,Alcohol} | {FBG_A1c_3Y} | 0.080 | 0.977 | 1.240 | 0.082 | 0.788 | 0.977 |
| 18 | {BP_2Y,BMI_2Y,LDL_3Y,Smoking,Alcohol,Exercise} | {FBG_A1c_3Y} | 0.089 | 0.975 | 1.238 | 0.091 | 0.788 | 0.975 |
| 19 | {BP_2Y,WC_2Y,FBG_A1c_3Y,Exercise} | {BMI_2Y} | 0.020 | 0.987 | 1.608 | 0.020 | 0.614 | 0.987 |
| 20 | {BP_2Y,WC_2Y,Smoking,Exercise} | {BMI_2Y} | 0.021 | 0.988 | 1.610 | 0.022 | 0.614 | 0.988 |
| 21 | {BP_2Y,LDL_3Y,FBG_A1c_3Y,Smoking,Alcohol,Diet} | {BMI_2Y} | 0.023 | 0.892 | 1.453 | 0.026 | 0.614 | 0.892 |
| 22 | {BP_2Y,WC_2Y,LDL_3Y,FBG_A1c_3Y,Smoking,Alcohol} | {BMI_2Y} | 0.080 | 0.987 | 1.607 | 0.082 | 0.614 | 0.987 |
| 23 | {BP_2Y,LDL_3Y,FBG_A1c_3Y,Smoking,Alcohol,Exercise} | {BMI_2Y} | 0.089 | 0.842 | 1.371 | 0.106 | 0.614 | 0.842 |
| 24 | {FBG_A1c_3Y,Diet,Exercise} | {Smoking} | 0.020 | 0.980 | 1.219 | 0.021 | 0.804 | 0.980 |
| 25 | {BP_2Y,Alcohol,Diet,Exercise} | {Smoking} | 0.021 | 0.982 | 1.221 | 0.021 | 0.804 | 0.982 |
| 26 | {BP_2Y,BMI_2Y,WC_2Y,Exercise} | {Smoking} | 0.021 | 0.969 | 1.205 | 0.022 | 0.804 | 0.969 |
| 27 | {BP_2Y,BMI_2Y,LDL_3Y,FBG_A1c_3Y,Alcohol,Diet} | {Smoking} | 0.023 | 0.987 | 1.227 | 0.023 | 0.804 | 0.987 |
| 28 | {BP_2Y,BMI_2Y,WC_2Y,LDL_3Y,FBG_A1c_3Y,Alcohol} | {Smoking} | 0.080 | 0.987 | 1.227 | 0.082 | 0.804 | 0.987 |
| 29 | {BP_2Y,BMI_2Y,LDL_3Y,FBG_A1c_3Y,Alcohol,Exercise} | {Smoking} | 0.089 | 0.984 | 1.223 | 0.090 | 0.804 | 0.984 |
| 30 | {BP_2Y,Smoking,Diet,Exercise} | {Alcohol} | 0.021 | 0.975 | 1.656 | 0.021 | 0.589 | 0.975 |
| 31 | {BP_2Y,BMI_2Y,LDL_3Y,FBG_A1c_3Y,Smoking,Diet} | {Alcohol} | 0.023 | 0.962 | 1.633 | 0.024 | 0.589 | 0.962 |
| 32 | {BP_2Y,BMI_2Y,WC_2Y,LDL_3Y,FBG_A1c_3Y,Smoking} | {Alcohol} | 0.080 | 0.821 | 1.393 | 0.098 | 0.589 | 0.821 |
| 33 | {BP_2Y,BMI_2Y,LDL_3Y,FBG_A1c_3Y,Smoking,Exercise} | {Alcohol} | 0.089 | 0.947 | 1.608 | 0.094 | 0.589 | 0.947 |
